# Complete plastome genomes of three medicinal heliotropiaceae species: comparative analyses and phylogenetic relationships

**DOI:** 10.1186/s12870-024-05388-8

**Published:** 2024-07-10

**Authors:** Mohammad S. Alawfi, Dhafer A. Alzahrani, Enas J. Albokhari

**Affiliations:** 1 Department of Biology, College of Sciences, King Khalid University, Abha, Saudi Arabia; 2 Department of Biological Sciences, Faculty of Sciences, King Abdulaziz University, Jeddah, Saudi Arabia; 3 Department of Biological Sciences, Faculty of Applied Sciences, Umm Al-Qura University, Makkah, Saudi Arabia

**Keywords:** Heliotropiaceae, Boraginales, Plastome, Phylogenetic tree

## Abstract

**Background:**

Heliotropiaceae is a family of the order Boraginales and has over 450 species. The members of the family Heliotropiaceae have been widely reported to be used in traditional medicine Over time, the classification of Heliotropiaceae has remained uncertain and has moved from family to subfamily, or conversely.

**Results:**

In the present study, we sequenced, analyzed, and compared the complete plastomes of *Euploca strigosa*, *Heliotropium arbainense*, and *Heliotropium longiflorum* with the genomes of related taxa. The lengths of the plastomes of *E. strigosa*, *H. arbainense*, and *H. longiflorum* were 155,174 bp, 154,709 bp, and 154,496 bp, respectively. Each plastome consisted of 114 genes: 80 protein-coding genes, 4 ribosomal RNA genes, and 30 transfer RNA genes. The long repeats analysis indicated that reverse, palindromic, complement and forward repeats were all found in the three plastomes. The simple repeats analysis showed that the plastomes of *E. strigosa*, *H. arbainense*, and *H. longiflorum* contained 158, 165, and 151 microsatellites, respectively. The phylogenetic analysis confirmed two major clades in the Boraginales: clade I comprised Boraginaceae, while clade II included Heliotropiaceae, Ehretiaceae, Lennoaceae, and Cordiaceae. Inside the family Heliotropiaceae, *E. strigosa* is nested within the *Heliotropium* genus.

**Conclusions:**

This study expands our knowledge of the evolutionary relationships within Heliotropiaceae and offers useful genetic resources.

**Supplementary Information:**

The online version contains supplementary material available at 10.1186/s12870-024-05388-8.

## Background

Heliotropiaceae (= Heliotropioideae) is a family of the order Boraginales and has over 450 species [1]. Most species of Heliotropiaceae are annual or perennial herbaceous plants, but there are also subshrubs, shrubs, lianas, or small trees. The leaves are alternate, simple, the inflorescence usually thyrsoid or scorpioid cyme, the flowers with 5 sepals and petals, five stamens, the ovary has two carpels, the fruit is dry or fleshy [1–3].

**Fig. 1:**
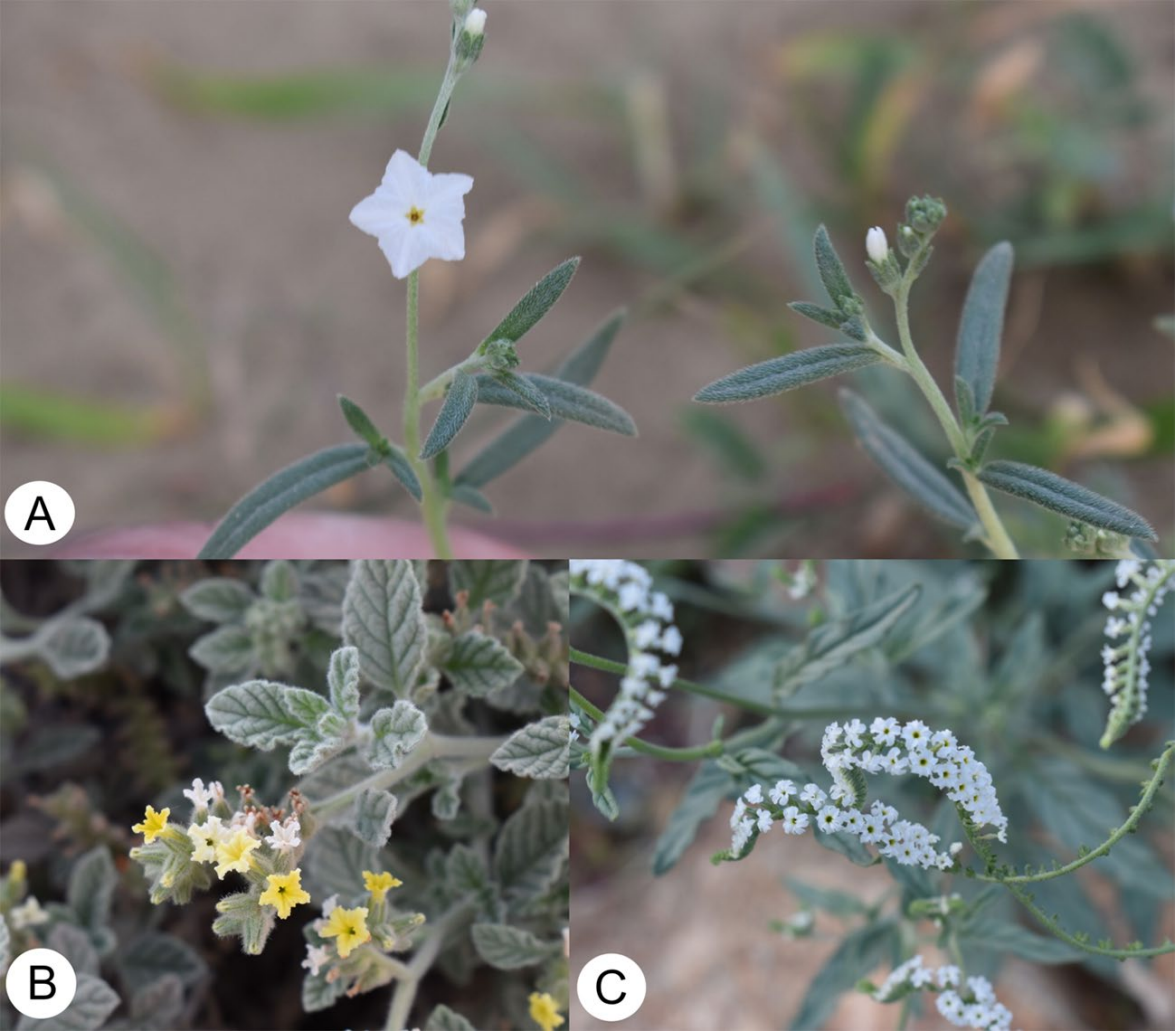
(**A**) leaves and flowers of *E. strigosa*, (**B**) leaves and flowers of *H. arbainense*, (**C**) leaves and flowers of *H. longiflorum*. Plants photos by M. Alawfi

The main source of modern pharmaceutical discoveries is traditional medicine, which is mostly based on the use of medicinal herbs [4, 5]. The herbal genomics studies will enhance and contribute to the discovery of genes controlling pharmaceutical traits [6]. For example, the identification of the precursor gene that is involved in the biosynthesis of lyciumins produced by *Lycium barbarum* has enabled scientists to identify different novel lyciumin chemotypes in other species from different families [7]. The members of the family Heliotropiaceae have been widely reported to be used in traditional medicine [8–10]. Species, such as *Euploca strigosa*,* Heliotropium arbainense* and *Heliotropium longiflorum* have been used to treat various diseases (Fig. 1). *E. strigosa* was used to treat gastrointestinal pain, gum boils, respiratory distress, sore eyes and vascular disorders [11, 12]; *H. arbainense* was reported to be effective in lower blood pressure and used as antimicrobial [13, 14]; *H. longiflorum* was used against cavities and in the treatment of allergies and febrile diseases [15, 16].

In the traditional taxonomy, the members of Heliotropiaceae were treated as subfamily Heliotropioideae within the Boraginaceae family [17–22]. The Angiosperm Phylogeny Group (APG) and some phylogenetic studies continue to support this classification [23–28]. In contrast, several phylogenetic studies have identified Heliotropiaceae as a distinct family in the Boraginales [1, 29–34]. All the studies that clarified the evolutionary relationships of the family Heliotropiaceae were based on a small number of nuclear DNA, plastome, and mitochondrial genes [35].

The intrafamilial classification of Heliotropiaceae remains uncertain. The family Heliotropiaceae was classified by Schrader in 1819 as comprising two genera: *Heliotropium* and *Tournefortia* [36]. Since then, several authors have suggested different classifications at the genus level, but these classifications have not been widely accepted [17, 37, 38]. In 1998, Förther divided Heliotropiaceae into eight genera: *Heliotropium*, *Tournefortia*, *Argusia*, *Ixorhea*, *Nogalia*, *Ceballosia*, *Hilgeria*, and *Schleidenia* [39]. In 2003, Hilger and Diane, based on *trnL* and ITS1 sequence data, recognized five genera within Heliotropiaceae: *Euploca*, *Heliothamnus*, *Heliotropium*, *Ixorhea*, and *Myriopus* [35]. These authors concluded, among others, that the *Euploca* species should be recognized as a separate genus from the *Heliotropium* genus. Moreover, the *Tournefortia* species has been transferred from the genus level to nest within the *Heliotropium* genus. Recently, the family has been widely classified into four genera: *Euploca*, *Heliotropium*, *Ixorhea*, and *Myriopus* [1].

The plastome offers valuable insights into evolutionary relationships between taxa [40]. The chloroplast is a cell organelle inside plant cells and performs the photosynthesis process [41]. The content, structure, and arrangement of genes in the plastome of flowering plants are extremely stable [42]. The plastome in angiosperm taxa has circular and quadripartite structures; however, recent research has revealed multibranched linear structures in several species of flowering plants [43]. The plastome is characterized by two identical copies of the inverted repeat (IR) separated by a small single-copy region (SSC) and a large single-copy region (LSC) [44]. The plastome sequence has been extensively used in phylogenetic studies; more than 5,998 plastomes of plants can be found in the National Center for Biotechnology Information (NCBI) database [45]. The utilization of plastome sequence can provide more reliable results for evolutionary relationships than using a few genes [46].

In this paper, we report the complete plastomes of three Heliotropiaceae taxa: *Euploca strigosa*, *Heliotropium arbainens*e, and *Heliotropium longiflorum*. The ultimate goals of this study were to: (i) obtain complete plastome genomes of *E. strigosa*, *H. arbainense*, and *H. longiflorum*, (ii) analyze and identify the features of genes, utilization of codons, RNA editing sites, and long and simple sequence repeat (SSR), IR junctions and sequence divergence, (iii) shed light on the intrafamilial classification of the family Heliotropiaceae and its evolutionary relationships with other families of the order Boraginales.

## Results

### Characteristics of*E. strigosa*,* H. arbainense*, and *H. longiflorum*.

**Fig. 2:**
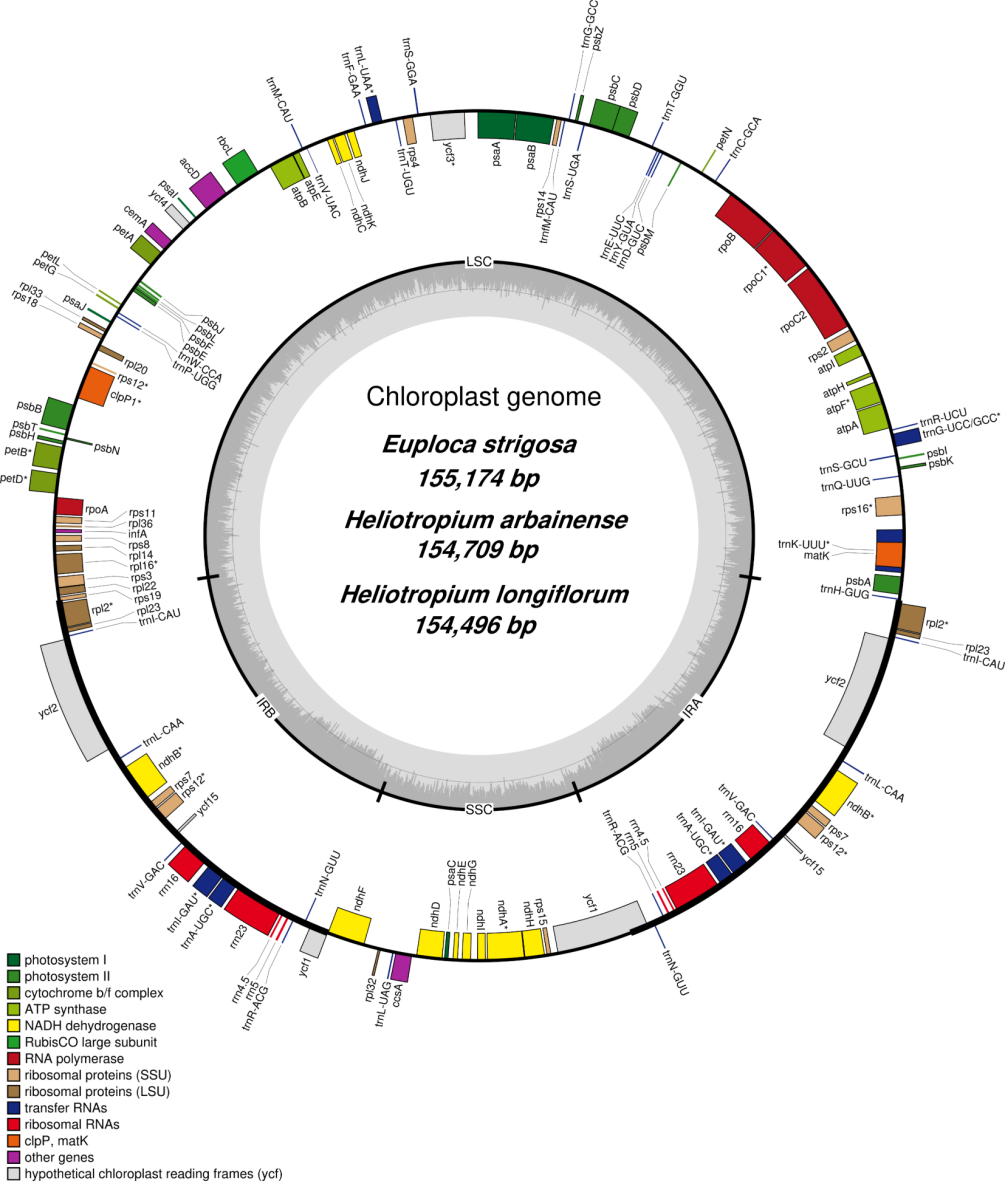
Plastome map of *E. strigosa*, *H. arbainense* and *H. longiflorum*. Genes found in the inside area of the circles are transcribed in a clockwise direction. Genes found in outside area of the circles are transcribed in anti-Clockwise direction. The colored bars identify functional genes. The LSC and SSC indicate the large and small single-copy regions. The IR indicate inverted repeat regions

The complete plastomes of *E. strigosa*,* H. arbainense*, and *H. longiflorum* were 155,174 bp, 154,709 bp, and 154,496 bp in size, respectively, with circular and quadripartite structures (Fig. 2 and Table S1). The plastome of *E. strigosa*,* H. arbainense*, and *H. longiflorum* contain the LSC region with lengths of 85,491 bp, 85,078 bp, and 84,742 bp, respectively; the SSC region with lengths of 17,979 bp, 17,995 bp, and 17,920 bp, respectively; and the IR regions with lengths of 25,852 bp, 25,818 bp, and 25,917 bp, respectively (Table S1). The overall GC content of *E. strigosa* was 37.8%, of *H. arbainense* was 37.70%, and of *H. longiflorum* was 37.41%. The IR regions occupied most of the GC contents, ranging from 43.10% in *E. strigosa* and *H. arbainense* to 42.95% in *H. longiflorum* (Table S1).

The plastomes of *E. strigosa*, *H. arbainense*, and *H. longiflorum* comprised 134 genes. Table S2 displays the 114 genes that were found in the three plastomes, which included 19 genes duplicated in IR regions (*trnI-CAU*,* trnL-CAA*,* trnV-GAC*,* trnl-GAU*,* trnA-UGC*,* trnR-ACG*,* trnN-GUU*,* rps7*,* rps12*,* rpl2*,* rpl23*,* ndhB*,* ycf2*,* ycf15*,* ycf1*,* rrn5*,* rrn4.5*,* rrn16* and *rrn23*). The *rps12* gene was duplicated in IR regions as well as in the LSC region. All plastomes included 4 rRNA genes, 30 tRNA genes, and 80 protein-coding genes. The SSC region included 1 tRNA gene and 12 protein-coding genes; the LSC region included 22 tRNA genes and 60 protein-coding genes; the IR regions included 4 rRNA genes, 7 tRNA genes, and 8 protein-coding genes. All three plastomes included introns in some protein-coding and tRNA genes (Table S3). A total of 17 (18 in *H. arbainense*) of the 114 genes contained introns, 15 genes (16 in *H. arbainense*) comprised 1 intron, and 2 genes (*ycf3* and *clpP1*) comprised 2 introns (Table S3). The *trnK-UUU* gene has the longest intron, with 2487 bp in *E. strigosa*, 2488 bp in *H. arbainense*, and 2472 bp in *H. longiflorum* (Table S3).

### Codon usage

**Fig. 3:**
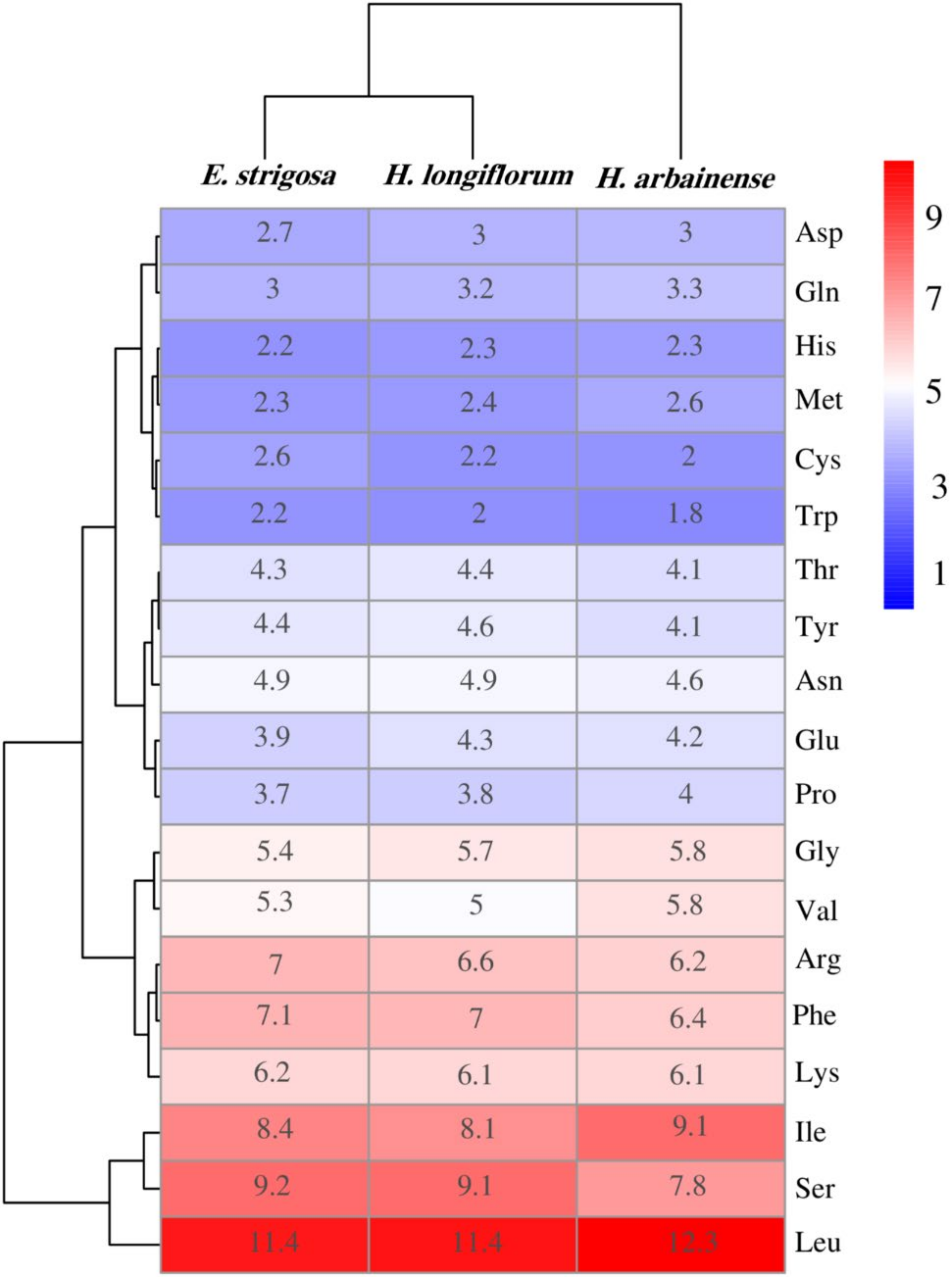
Codon preference heat map of *E. strigosa*, *H. arbainense* and *H. longiflorum* plastomes. The RSCU values of amino acids were used as the basis for tree clustering. As the red colour deepens, the RSCU value increases. As the blue colour deepens, the RSCU value decreases

The codon usage frequency of protein-coding genes and tRNA genes was examined in the three plastomes; lengths were 81,762 bp in *E. strigosa*, 82,390 bp in *H. arbainense*, and 81,448 bp in *H. longiflorum*. The plastome of *E. strigosa* was encoded by 27,254 codons; leucine had the most codons (11.42%), while tryptophan was the least common amino acid (2.26%) (Fig. 3). A total of 28 codons had relatively synonymous codon usage (RSCU) greater than 1, while 34 codons had less than 1 (Table S4). The plastome of *H. arbainense* was encoded by 27,463 codons; leucine had the most codons (12.36%), while tryptophan was the least common amino acid (1.86%) (Fig. 3). A total of 29 codons had relatively synonymous codon usage (RSCU) greater than 1, while 33 codons had less than 1 (Table S5). The plastome of *H. longiflorum* was encoded by 27,148 codons; leucine had the most codons (11.49%), while tryptophan was the least common amino acid (2.00%) (Fig. 3). A total of 28 codons had relatively synonymous codon usage (RSCU) greater than 1, while 34 codons had less than 1 (Table S6). All amino acids in the three plastome reflected codon usage bias, with the exception of tryptophan and methionine, which contained RSCU values equal to 1.

### RNA editing sites

**Fig. 4:**
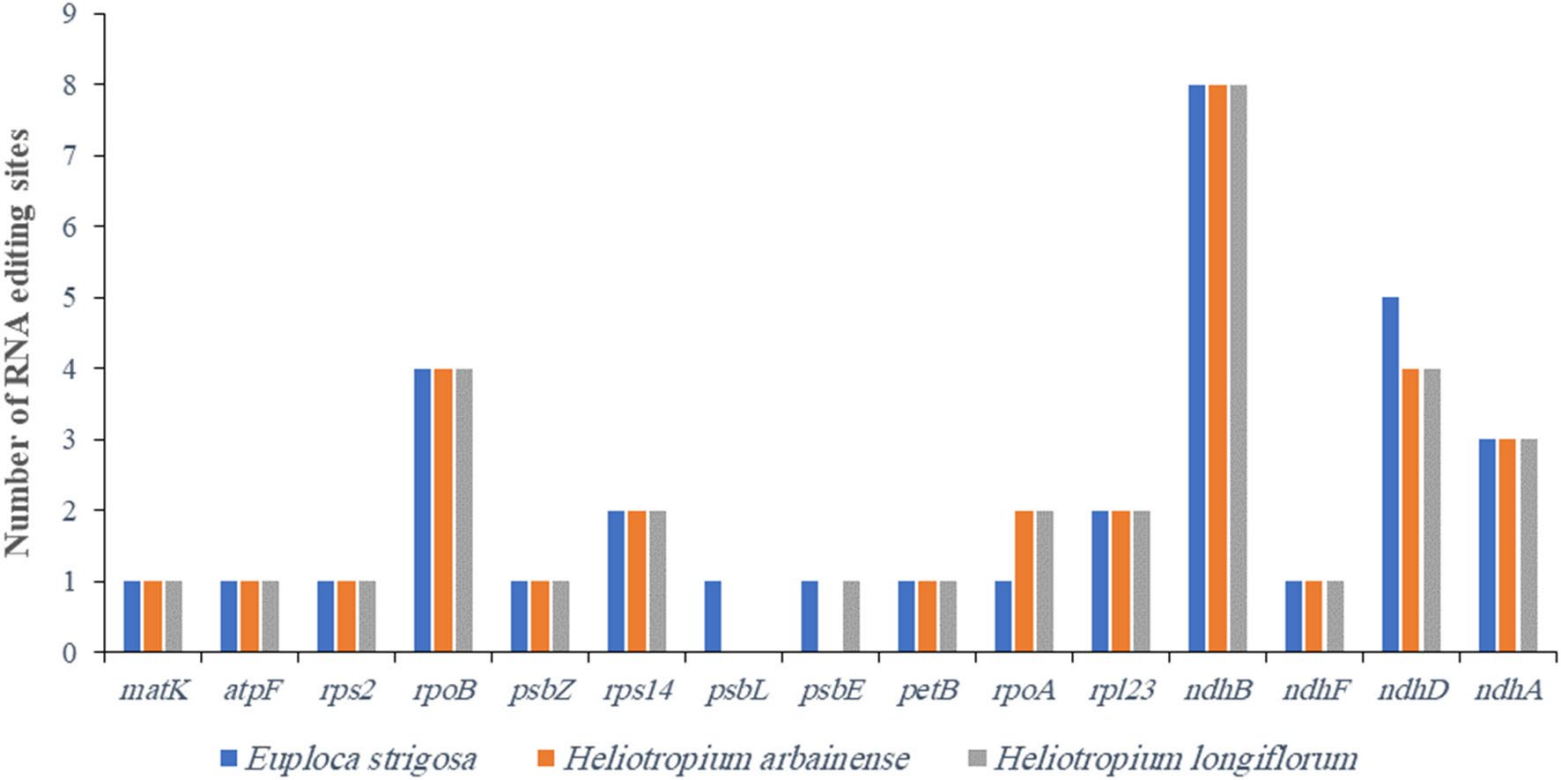
Predicted C-to-U RNA editing sites in *E. strigosa*, *H. arbainense* and *H. longiflorum* plastomes

Using the PREPACT Tool, the C-to-U RNA editing sites in the plastomes of *E. strigosa*,* H. arbainense*, and *H. longiflorum* have been predicted. The analysis identified 34 editing sites in *E. strigosa*, 32 in *H. arbainense*, and 33 in *H. longiflorum* (Fig. 4). The RNA editing sites were found in 14 to 16 protein-coding genes in the three plastomes (*atpF*,* ndhF*,* ndhD*,* ndhB*,* ndhA*,* matK*,* psbE*,* petB*,* psbL*,* psbZ*,* rpoC1*,* rpoB*,* rpoA*,* rpl23*,* rps2*, and *rps14*) (Fig. 4 and Tables S7 and S8). In *E. strigosa*, 88.23% of the editing sites were present in the next nucleotide of the codon and 11.77% were in the first nucleotide (Table S7). In *H. arbainense*, 90.62% of the editing sites were present in the next nucleotide of the codon and 9.38% were in the first nucleotide (Table S88). In *H. longiflorum*, 87.87% of the editing sites were present in the next nucleotide of the codon and 12.13% were in the first nucleotide (Table S8). The result also revealed that most amino acid conversions were from serine to leucine within the three plastomes (Tables S7 and S8).

### The long and simple sequence repeats

**Fig. 5:**
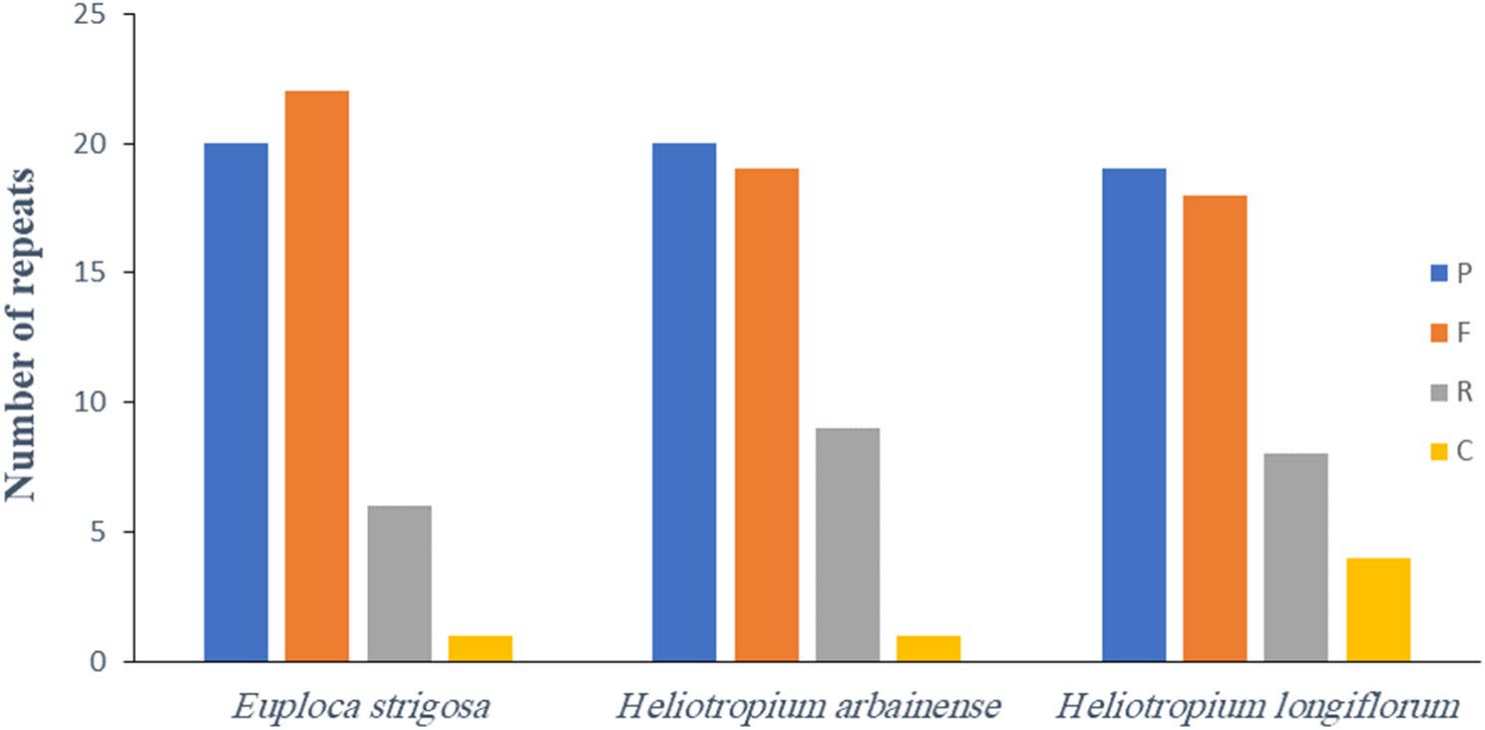
The number of different repeats in the plastomes of *E. strigosa*, *H. arbainense* and *H. longiflorum*. P = palindromic, F = forward, R = reverse and C = complement

The long repeats in *E. strigosa*,* H. arbainense*, and *H. longiflorum* plastomes were detected by the REPuter program. The results showed that the reverse, palindromic, complement and forward repeats were found in all plastomes, with 49 repeats found in all three genomes (Fig. 5). More specifically, analysis of *E. strigosa*,* H. arbainense*, and *H. longiflorum* recognized 1, 1, and 4 complement repeats, respectively; 22, 19, and 18 forward repeats, respectively; 20, 20, and 19 palindromic repeats, respectively; and 6, 9, and 8 reverse repeats, respectively (Fig. 5 and Tables S9, S10, and S11).

Most of the repeat sizes in *E. strigosa* were between 18 and 22 bp (73.46%), 23 and 29 bp (22.44%), and 40 to 44 bp (4.10%) (Table S9). In *H. arbainense*, the most of the repeat were between 18 and 22 bp (81.63%), 24 and 26 bp (14.29%), and 29 and 40 bp (4.08%) (Table S10). In *H. longiflorum*, most of the repeat sizes were between 18 and 23 bp (79.59%), 26 and 29 bp (12.24%), and 38 and 48 bp (8.17%) (Table S11). The intergenic spacer regions in *E. strigosa*,* H. arbainense*, and *H. longiflorum* harbored 54.08%, 51.03%, and 53.07% of repeats, respectively; the protein-coding genes harbored 31.63%, 35.71%, and 35.71% of repeats, respectively; and the tRNA genes harbored 14.29%, 13.26%, and 11.22% of repeats, respectively (Tables S9, S10, and S11).

Microsatellites, also known as simple sequence repeats (SSRs), are spread across the three plastomes. The plastomes of *E. strigosa*,* H. arbainense*, and *H. longiflorum* contained 158, 165, and 151 microsatellites, respectively (Tables S12, S13, and S14). In the plastome of *E. strigosa*, mononucleotides (A/T) harbored the majority of SSRs with 142 microsatellites (Table 1). Moreover, one dinucleotide (AT/AT), one trinucleotide (AAT/ATT), and two tetranucleotides (AAAC/GTTT and AAAG/CTTT). In the plastome *H. arbainense*, mononucleotides (A/T) harbored the majority of SSRs with 151 microsatellites (Table 1). Moreover, one dinucleotide (AT/AT), four tetranucleotides (AAAC/GTTT, AAAG/CTTT, AAAT/ATTT, and AATT/AATT), and one pentanucleotide (AAAAT/ATTTT). In the plastome *H. longiflorum*, mononucleotides (A/T) harbored the majority of SSRs with 133 microsatellites (Table 1). Moreover, one dinucleotide (AT/AT), two trinucleotides (AAG/CTT and AGC/CTG), two tetranucleotides (AAAC/GTTT and AAAT/ATTT), and one hexanucleotide (AAAAAG/CTTTTT).

**Fig. 6:**
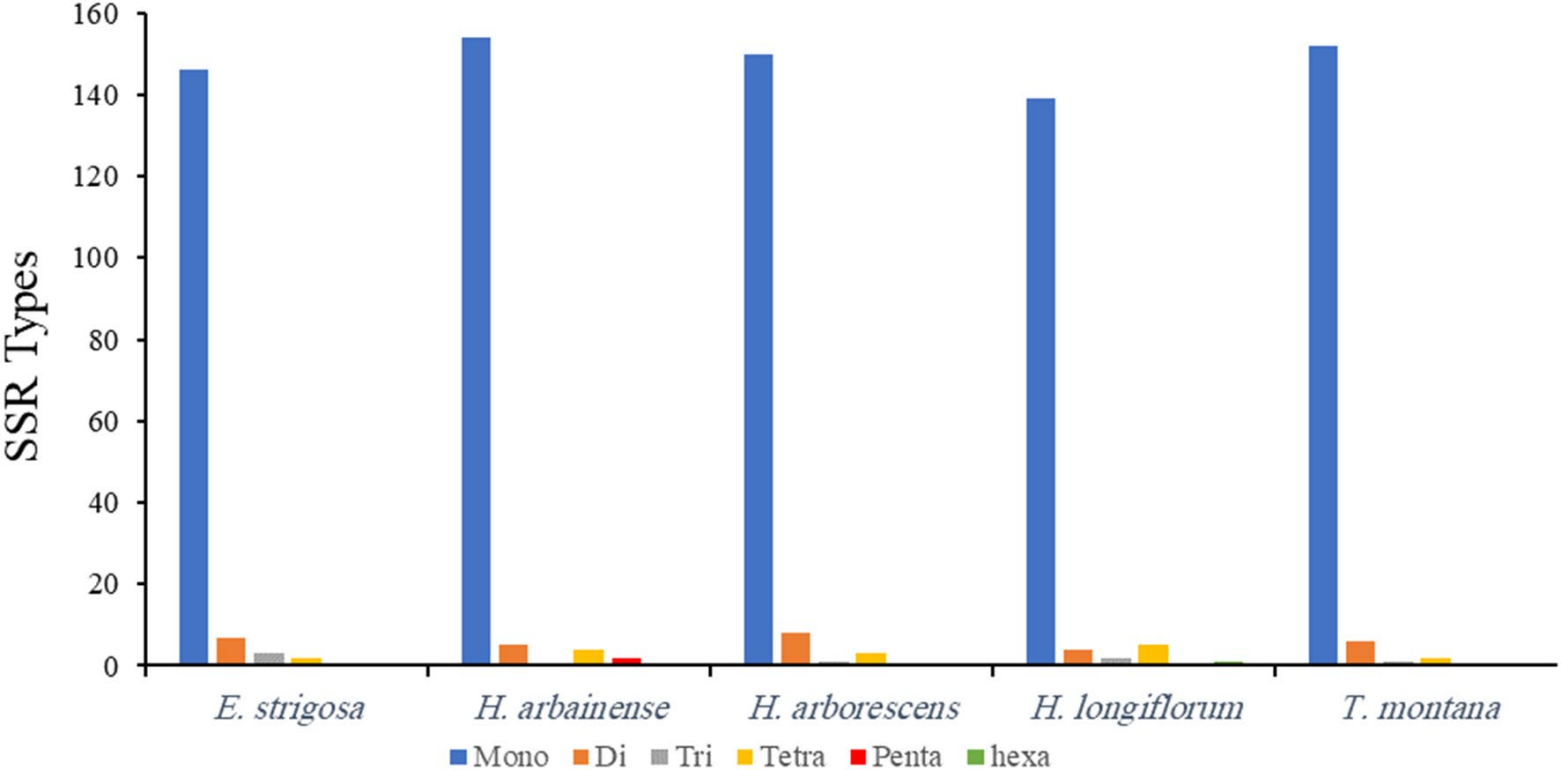
Number and types of SSR in five plastomes of Heliotropiaceae species

Another comparative analysis of the microsatellites was conducted between the *E. strigosa*,* H. arbainense*, and *H. longiflorum* plastomes and the other Heliotropiaceae plastomes available in the GenBank database (*H. arborescens* and *T. montana*). The results showed microsatellites types ranging from mononucleotide to hexanucleotide repeats (Fig. 6). The mononucleotide, dinucleotide, and tetranucleotide repeats were detected in all plastomes, trinucleotide repeats were found in all species except *H. arbainense*, pentanucleotide repeats were found only in *H. arbainense*, and hexanucleotide repeats were found only in *H. longiflorum* (Fig. 6).

**Table 1:** The microsatellites in plastomes of *E. strigosa*, *H. arbainense* and *H. longiflorum*

SSR type	Repeat unit	Species		
		*E*. *strigosa*	*H. arbainense*	*H. longiflorum*
Mono	A/T C/G	142 4	151 3	133 6
Di	AT/AT	7	5	4
Tri	AAG/CTT AAT/ATT AGC/CTG	0 3 0	0 0 0	1 0 1
Tetra	AAAC/GTTT AAAG/CTTT AAAT/ATTT AATT/AATT	1 1 0 0	1 1 1 1	2 0 3 0
Penta	AAAAT/ATTTT	0	2	0
Hexa	AAAAAG/CTTTTT	0	0	1

### Comparative analyses

**Fig. 7:**
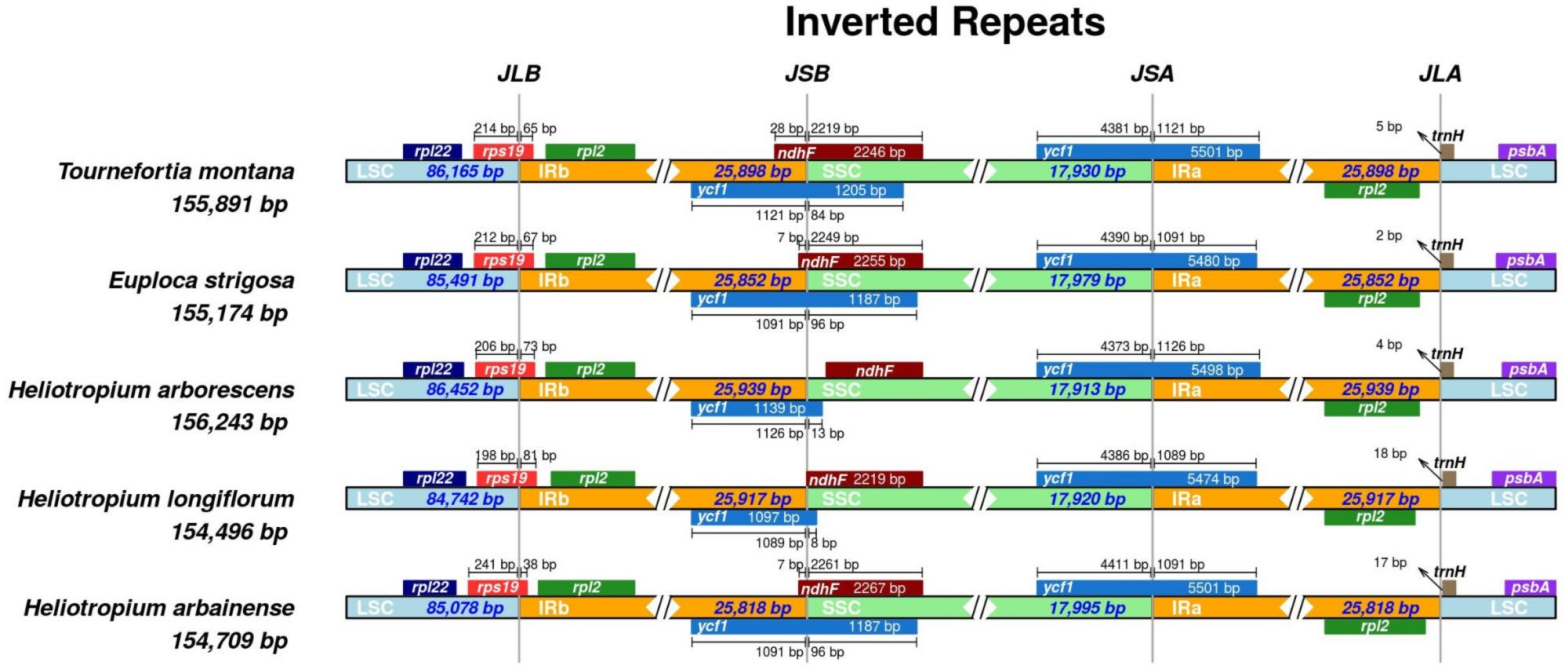
Comparison of the borders of the IR, SSC and LSC regions between the five plastomes of Heliotropiaceae species

The IR-SSC and IR-LSC boundaries of the plastomes of five Heliotropiaceae species (*E. strigosa*,* H. arbainense*,* H. arborescens*, *H. longiflorum*, and *T. montana*) were compared in this study. The analysis showed similarities among the five plastomes (Fig. 7). *H. arborescens* harbored the largest plastomes (156,243 bp), followed by *T. montana* (155,891 bp), *E. strigosa* (155,174 bp), *H. arbainense* (154,709 bp), and *H. longiflorum* (154,496 bp).

In addition, the analysis indicated that the *rpsl9* gene was located within the LSC and IRb boundaries in all plastomes (Fig. 7). The *ycf1* gene was present at the boundaries of the IRb/SSC regions in all plastomes: 1091 bp/96 bp in *E. strigosa*, 1091 bp/96 bp in *H. arbainense*, 1126 bp/13 bp in *H. arborescens*, 1089 bp/8 bp in *H. longiflorum*, and 1121 bp/84 bp in *T. montana.* Moreover, *ycf1* was located at the boundaries of the SSC/IRa regions in all plastomes: 4390 bp/1091 bp in *E. strigosa*, 4411 bp/1091 bp in *H. arbainense*, 4373 bp/1126 bp in *H. arborescens*, 4386 bp/1089 bp in *H. longiflorum*, and 4381 bp/1121 bp in *T. montana.* The *ndhF* gene was found only at the SSC region in *H. arborescens* and *H. longiflorum*, with 2234 bp and 2219 bp in length, respectively, while it was located at the boundaries of the IRb/SSC regions in the other taxa: 2249 bp/7 bp in *E. strigosa*, 2261 bp/7 bp in *H. arbainense*, and 2219 bp/28 bp in *T. montana*. No genes were found at the boundaries of IRa/LSC. The *psbA* and *trnH* genes were located totally in the LSC region in all plastomes.

### Divergence of protein-coding gene sequences

**Fig. 8:**
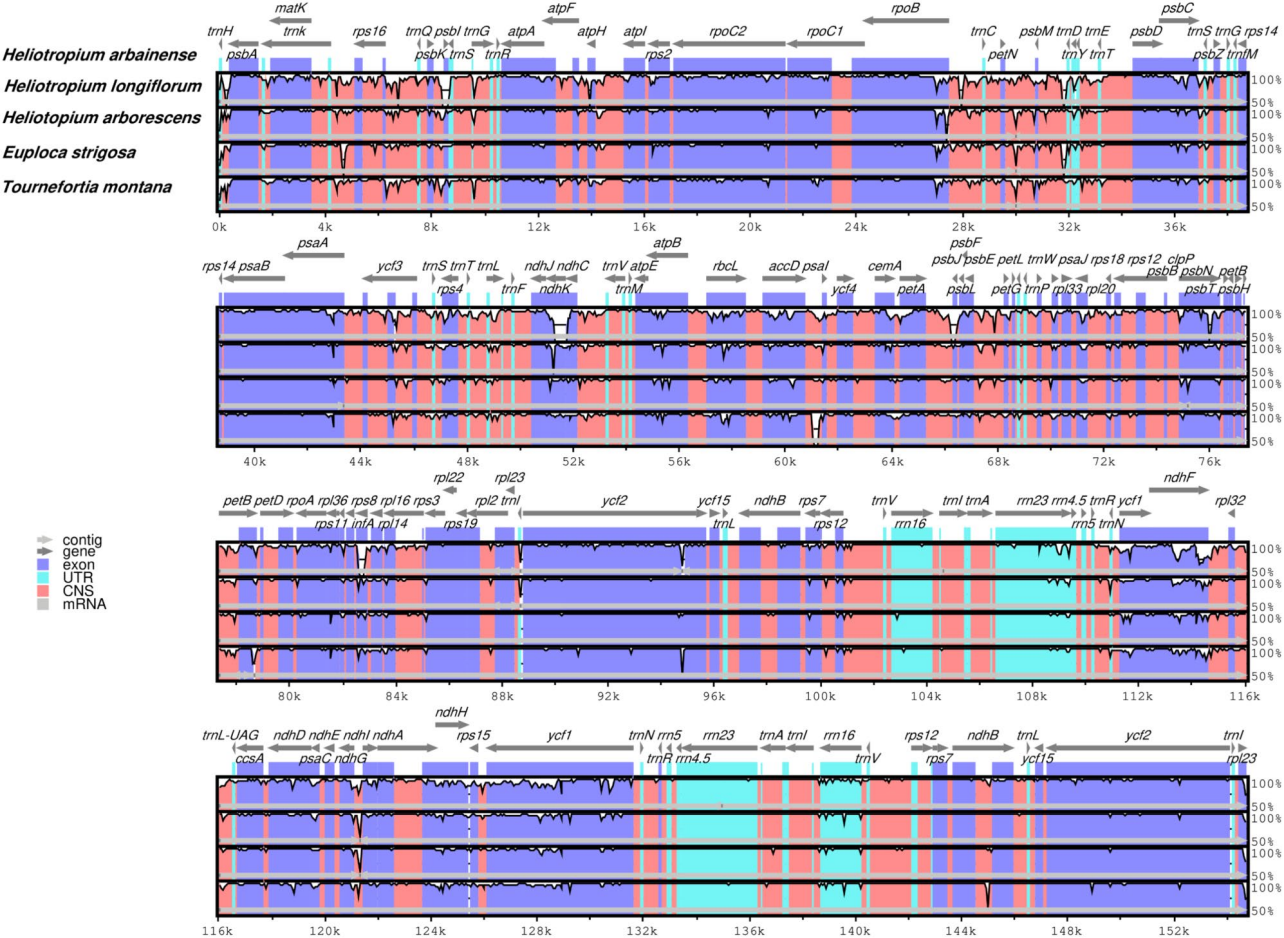
Five Heliotropiaceae plastomes were visually aligned using *H. arbainense* as a reference. The plastome coordinate is shown by the x-axis, while the identity percentage (between 50% and 100%) is represented by the y-axis. The direction of each gene is indicated by the upper arrows. CNS stands for conserved non-coding regions; UTR stands for untranslated regions. The mVISTA program was used for the sequence alignment

Five Heliotropiaceae plastomes were compared using the *H. arbainense* plastome as a reference. This was carried out in order to observe the sequence divergence regions (Fig. 8). The analysis revealed that all plastomes were extremely conserved, with few variable regions. Most sequence divergence was detected in noncoding regions rather than in the coding regions (Fig. 8). The *atpA*,* matK*,* rpoC1*,* rpoC2*,* rpoB*,* psbC*,* psaB*,* psaA*,* accD*,* clpP*,* rpoA*,* ycf2*,* ndhF*,* ndhH*, and *ycf1* genes had the highest divergence in the coding regions (Fig. 8).

### Characterization of substitution rates

**Fig. 9:**
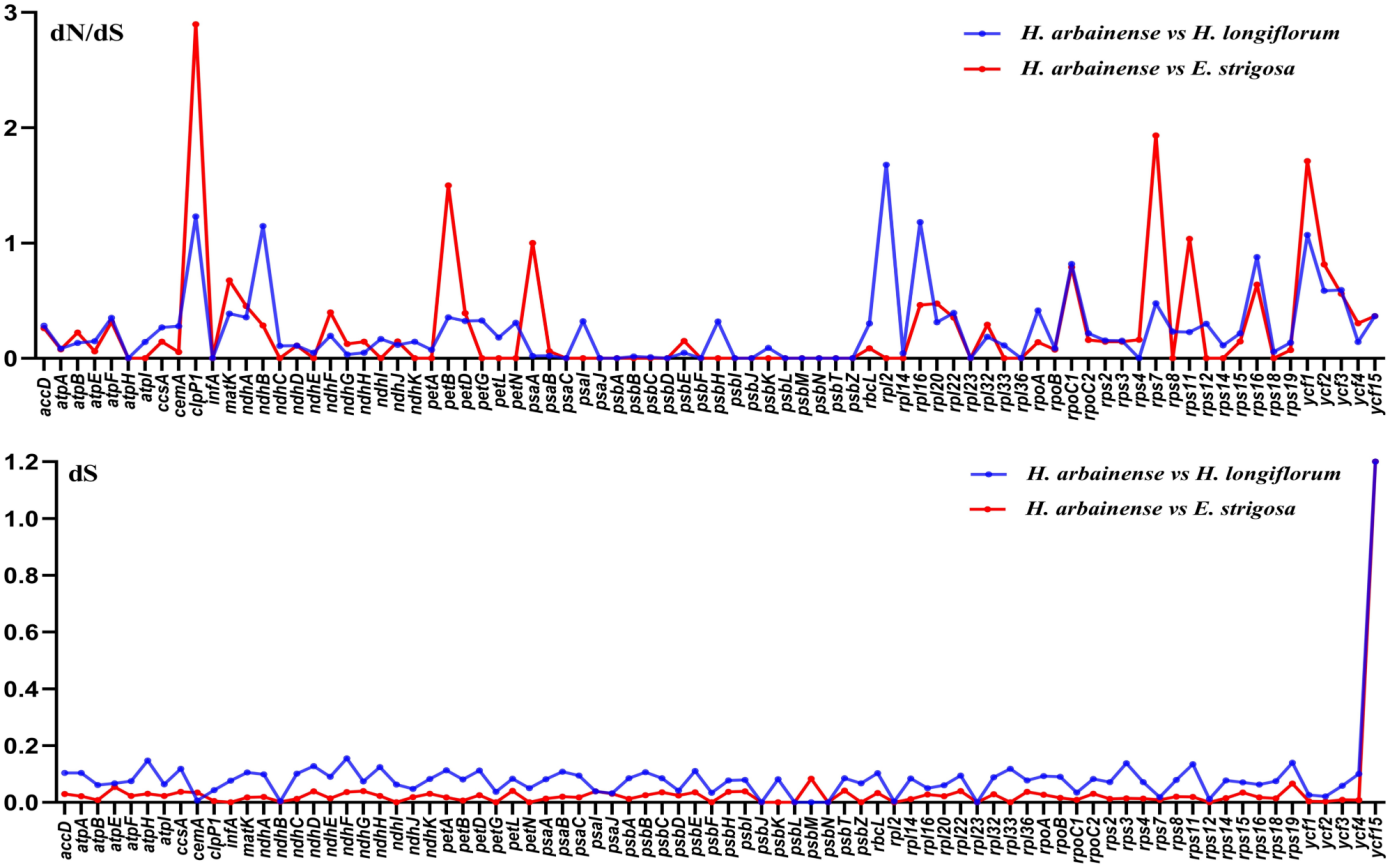
The synonymous (dS) subsituation and dN/dS ratio values of protein-coding genes from *H. arbainense* against *H. longiflorum* and *E. strigosa* plastomes

To identify the selective pressure within 80 protein-coding genes of three Heliotropiaceae plastomes, the rates of synonymous (dS) as well as the dN/dS ratio were computed. First, in comparing *H. arbainense* with *H. longiflorum*, several genes (*clpP1*,* ndhB*,* rpl2*,* rpl16*, and *ycf1*) were under selective pressure with dN/dS values > 1 (Fig. 9). Second, in comparing *H. arbainense* with *E. strigosa*, a number of genes (*clpP1*,* petB*,* psaA*,* rps7*,* rps11*, and *ycf1*) were also under selective pressure, with dN/dS values > 1 (Fig. 9). In both analyses, most dS values were < 1 in all genes, except in *ycf15* genes, which had dS values of 1.2 (Fig. 9).

### Nucleotide diversity (pi) analysis

**Fig. 10:**
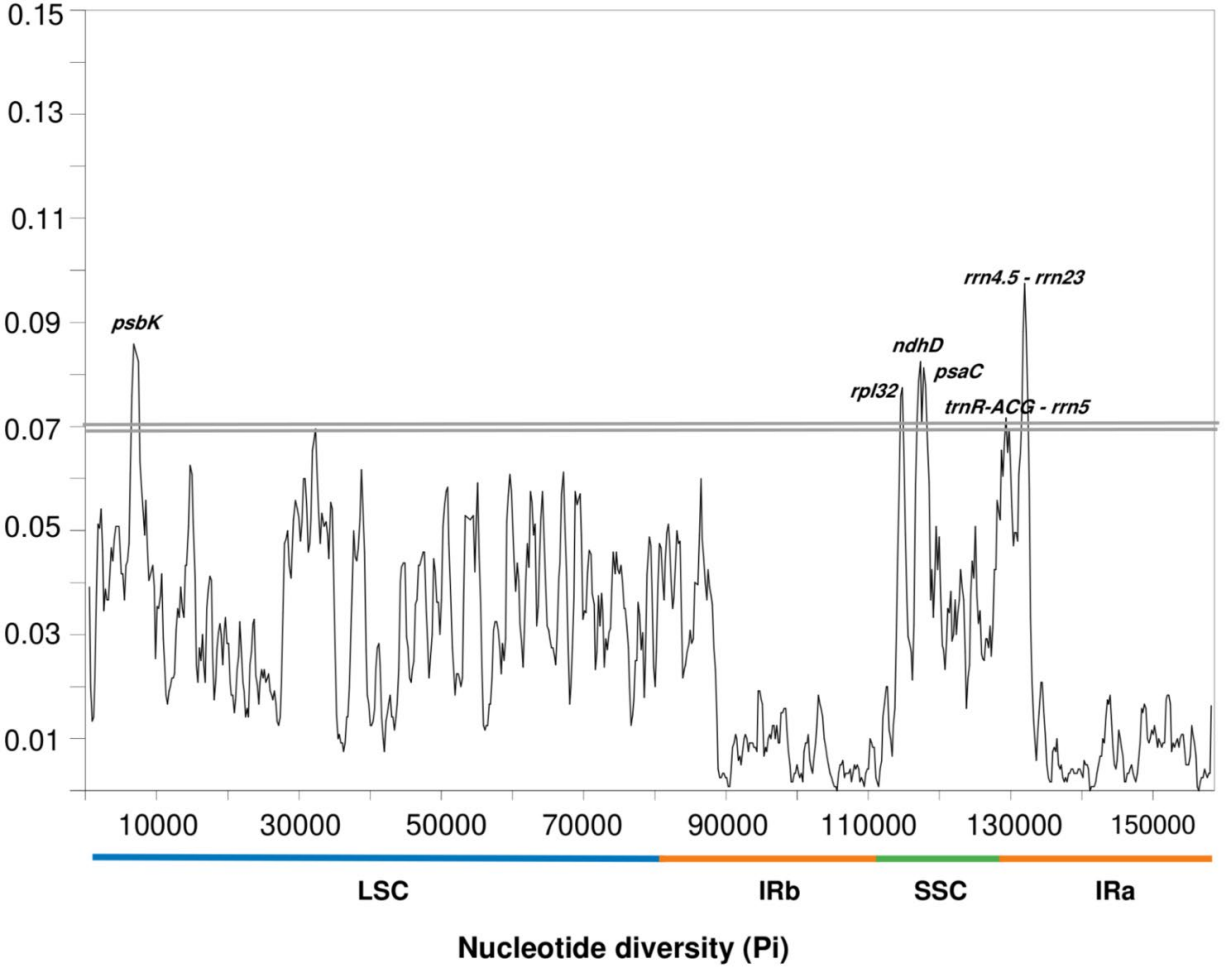
Nucleotide diversity values among *H. arbainense*, *H. longiflorum* and *E. strigosa* plastomes. Variation hotspots (Pi > 0.07) are labelled above the corresponding gene position

The sliding window analysis of nucleotide diversity (Pi) recognized several highly variable regions among the *E. strigosa*, *H. arbainense*, and *H. longiflorum* plastomes. As shown in Fig. 10, the range of nucleotide diversity (Pi) was found from 0.00000 to 0.09750. The nucleotide diversity in SSC and IRs regions is substantially higher than that in LSC region. Six sequence mutation hotspots (Pi > 0.07) were identified, of which one was placed in the LSC region (*psbK*), three were located in the SSC region (*rpl32*, *ndhD* and *psaC*), and two were found in the IRa region (*trnR-ACG* - *rrn5* and *rrn4.5* - *rrn23*). The highest degree of nucleotide diversity in the coding region and non-coding region was *psbK* and *rrn4.5* - *rrn23*, respectively.

### Phylogenetic analysis

**Fig. 11:**
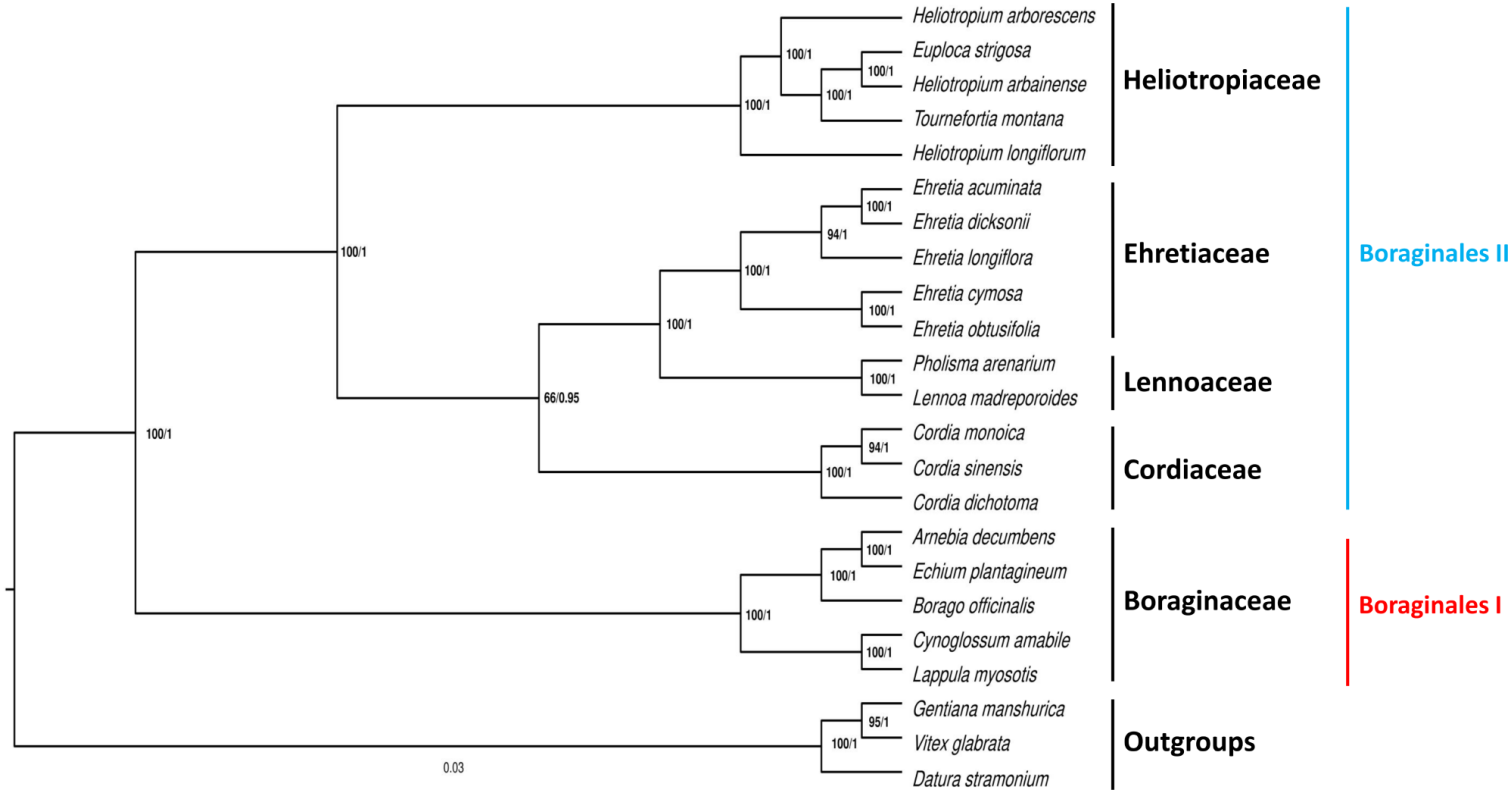
A phylogenetic tree showing the relationships between five families of the order Boraginales was produced by ML and BI analyses using 23 plastomes. The branch nodes numbers represent the (BS)/(PP) values

ML and BI phylogenetic analyses resulted in virtually identical phylogenetic trees. The results are presented as one tree indicating the support values of key nodes using Bootstrap (BS) and posterior probability (PP) values (Fig. 11). The order Boraginales fell into two clades: The first clade (Boraginales I) comprises Boraginaceae and the second clade (Boraginales II) comprises Heliotropiaceae, Cordiaceae, Lennoaceae, and Ehretiaceae. In the Boraginales I clade, the family Boraginaceae comprises two subfamilies: Cynoglossoideae and Boraginoideae with strong supported values (BS = 100/PP = 1).

In the Boraginales II clade, Ehretiaceae and Lennoaceae resolved as sisters with strong supported values (BS = 100/PP = 1). Cordiaceae was found to be the immediate sister to Ehretiaceae and Lennoaceae, but with strong support only from PP (BS = 66/PP = 0.95). Heliotropiaceae was the first clade to diverge in the Boraginales II clade and was sister to the three families with strong supported values (BS = 100/PP = 1). Inside the family Heliotropiaceae, *E. strigosa* and *H. arbainense* resolved as sisters (Fig. 11), with strong supported values (BS = 100/PP = 1). *T. montana* was found to be the immediate sister of *E. strigosa* and *H. arbainense*, with strong supported values (BS = 100/PP = 1). *H. longiflorum* and *H. arborescens* were the first and second species to diverge in the Heliotropiaceae clade.

## Discussion

The complete plastome presents an abundance of genetic information and markers that enable scientists to understand the complicated evolutionary history of land plants [47]. In this article, we report the plastomes of three taxa belonging to the family Heliotropiaceae. The plastomes of *E. strigosa*, *H. arbainense*, and *H. longiflorum* were structurally similar to the plastomes of other Boraginales species [48–50]. The plastome sizes were 155,174 bp in *E. strigosa*, 154,709 bp in *H. arbainense*, and 154,496 bp in *H. longiflorum* (Fig. 2). The plastomes of *E. strigosa*, *H. arbainense*, and *H. longiflorum* had GC contents of 37.80%, 37.70%, and 37.41%, respectively (Table S1). The variance in GC content across several taxa within same genus might be caused by the varying codon usage biases among taxa. The highest GC contents were found within IR regions, with 43.10% in *E. strigosa* and *H. arbainense*, and 42.95% in *H. longiflorum* (Table S1), which was greater than that of the SSC and LSC regions, possibly as a result of the presence of all rRNAs in these regions of the plastome [51]. Considering that they possess greater GC than the LSC and SSC regions, the IR regions might be more stable [52]. The three plastomes contained 114 genes (including 19 genes duplicated in IR regions), and split into 80 protein coding genes, 30 tRNA genes, and 4 rRNA genes (Table S2). Introns were identified in all plastomes (Table S3). Intron content is extremely conserved in the plastomes of angiosperms [53], which is essential for regulating gene expression [54].

The codon usage analysis indicated that all genes in the three plastomes were encoded by 27,254 codons in *E. strigosa*, 27,463 codons in *H. arbainense*, and 27,148 codons in *H. longiflorum*. Codon use is essential for gene expression [55], and it has been linked to gene expression level, amino acid conservation, transcriptional preference, and GC content [56]. Most codons were coded for leucine (Fig. 3). The majority of codons in all three plastomes had an RSCU value of less than 1 (Table S4), similar to the results for *H. arborescens* [57]. The C-to-U RNA editing sites analysis predicted 34 editing sites in *E. strigosa*, 32 in *H. arbainense*, and 33 in *H. longiflorum* that were distributed within 14 to 16 protein-coding genes among the three species (Fig. 4 and Tables S7 and S8). The RNA editing is a crucial aspect of the alteration of nucleotides in the mRNA of genes with functions within the plastome [58]. Most amino acid conversions were found to be serine to leucine, as noted in most angiosperm plants [47, 59].

The long repeat sequence analysis of *E. strigosa*, *H. arbainense*, and *H. longiflorum* recognized the forward and palindromic repeats were the most common repeats (Fig. 5 and Tables S9, S10, and S11), as found in the most angiosperm plastomes [60–64]. The number and regions of repeat sequences might influence the recombination and arrangement processes within the plastome [65]. The SSR analysis demonstrated that the plastomes of *E. strigosa*, *H. arbainense*, and *H. longiflorum* comprised 158, 165, and 151 microsatellites, respectively (Table 1). It has been proven that the SSRs are significant molecular markers in taxonomic studies [66]. Additionally, they have contributed to other research fields, such as the analysis of gene flow and the determination of genetic variation across plant genomes [67, 68]. The majority of SSRs were mononucleotides (Fig. 6), with A/T repeats representing the most frequent type, as noted in most plastomes of angiosperm [66, 69].

The IR-SSC and IR-LSC boundaries between the five plastomes of Heliotropiaceae were compared (Fig. 7). Variations in plastome size have been linked to the expansion and contraction of IR regions [70, 71]. The results indicated that most of the genes found at the junctions of Heliotropiaceae plastomes were well conserved, except for the *ndhF* gene which was found at IRb/SSC regions in *E. strigosa*, *H. arbainense* and *T. montana* were entirely in the SSC region in *H. arborescens* and *H. longiflorum*. The unstable location of *ndhF* gene has been noted in Boraginales species, for example, it’s found at IRb/SSC junctions in *Arnebia euchroma*, *Trigonotis peduncularis* and *Nonea vesicaria*, and entirely in the SSC region in *Ehretia dicksonii*, *Cynoglossum amabile* and *Lappula myosotis* [57].

Analysis of the sequence divergence region of the five plastomes of Heliotropiaceae detected that all plastomes were extremely conserved; however, a few of variable regions were found in *matK*,* atpA*,* rpoC2*,* rpoC1*,* rpoB*,* psbC*,* psaB*,* psaA*,* accD*,* clpP*,* rpoA*,* ycf2*,* ndhF*,* ndhH*, and *ycf1* genes (Fig. 8). These divergence markers, which have been extensively used in phylogenetic studies of angiosperms [72–74]. It would be useful to utilize these high diversity regions as species-specific DNA barcoding in the Heliotropiaceae plastomes. The results of the selective pressure rate analysis of 80 protein-coding genes among *E. strigosa*, *H. arbainense*, and *H. longiflorum* revealed that the dN/dS ratio was below 1 in most genes, except the *clpP1*,* ndhB*,* rpl2*,* rpl16*, and *ycf1* genes, which were found under positive selection and had dN/dS ratios greater than 1 (Fig. 9). These genes functions need additional investigation because they may be important in the adaptive evolution of Heliotropiaceae taxa.

The nucleotide diversity analysis recognized six mutated hotspots (Fig. 10), and some of them can also be observed in other angiosperms, such as *psbK*,* rpl32*, *ndhD* and *psaC* [75–77]. These regions are expected to have an increase in the substitution of nucleotides, which will make them valuable references for the use as DNA barcodes at the species level. Moreover, the plastome can be considered as a super barcode for species identification because it is hundreds of times longer than the common barcode sequence and has a lot of variation sites [78]. The identified mutation hotspots in this analysis are promising molecular markers, which can provide several informative sites for the molecular identification and phylogeny of the Heliotropiaceae family.

According to the results of phylogenetic analysis, there are two main clades within the order Boraginales (Boraginales I and Boraginales II) (Fig. 11), consistent with the results of previous studies [1, 31]. The first clade comprises Boraginaceae with two subfamilies (Cynoglossoideae and Boraginoideae), consistent with the results of prior studies [57, 79]. The second clade comprises four families: Heliotropiaceae, Cordiaceae, Lennoaceae, and Ehretiaceae, as inferred in various phylogenetic analyses of Boraginales [32, 33, 57, 80].

The results of the infrafamilial relationships of Heliotropiaceae show that *T. montana* nested in the *Heliotropium* genus (Fig. 11), consistent with a number of phylogenetic analyses [1, 30, 33, 80], and here we agree with suggestions to transfer *Tournefortia* taxa to the *Heliotropium* genus [81, 82]. Moreover, the analysis shows that *E. strigosa* nested in the *Heliotropium* genus and was sister to *H. arbainense.* Traditionally, the *Euploca* genus (previously *Heliotropium* section *Orthostachys*) has been recognized as part of the *Heliotropium* genus [17, 37–39]. Moreover, no single morphological characteristics can be used to distinguish all *Euploca* species from the *Heliotropium* genus [83]. In 2003, based on *trnL* and ITS1 sequence data, Hilger and Diane recognized *Euploca* as a separate genus in Heliotropiaceae [35]. However, the study relied on a limited number of *Euploca* taxa (*Heliotropium* section *Orthostachys*) [83] Moreover, in 2005, Craven rejected this taxonomic separation, suggesting that the entire Heliotropiaceae family is composed of a single large genus [81]. Our results favor expanding the *Heliotropium* genus to include all members of *Euploca* and *Tournefortia*.

## Conclusion

In this study, the basic characteristics of three plastomes from the Heliotropiaceae family (*E. strigosa*,* H. arbainense* and *H. longiflorum*) were analyzed and compared. The base composition, long repeats, SSRs, codon usage, IR boundaries, RNA editing sites, sequence divergence regions, characterization of substitution rates and nucleotide diversity (Pi) were analyzed and identified in these plastomes. The plastome sizes of the three Heliotropiaceae species were ranging from 155,174 bp to 154,496 bp, most codons were coded for leucine, the C-to-U RNA editing sites ranged from 34 to 32 editing sites, the forward and palindromic repeats were the most common long repeats and the majority of SSRs were mononucleotides (A/T repeats). In the three Heliotropiaceae plastomes, the *ndhF* gene showed an unstable location at the junctions, while *matK*, *atpA*, *rpoC2*, *rpoC1*, *rpoB*, *psbC*, *psaB*, *psaA*, *accD*, *clpP*, *rpoA*, *ycf2*, *ndhF*, *ndhH* and *ycf1* genes were the most variable regions, the dN/dS ratio was above 1 in *clpP1*, *ndhB*, *rpl2*, *rpl16*, and *ycf1* genes, several mutated hotspots were recognized such as *psbK*, *rpl32*, *ndhD* and *psaC* genes. In phylogenetic analysis, two major clades were recognized within the order Boraginales. The first clade comprised one family, Boraginaceae, and the second clade included four families: Heliotropiaceae, Lennoaceae, Ehretiaceae, and Cordiaceae. The findings regarding the infrafamilial relationships of Heliotropiaceae indicated that *Euploca* and *Tournefortia* taxa nested in the *Heliotropium* genus. The authors of this paper favor expanding the *Heliotropium* genus to include all members of *Euploca* and *Tournefortia.* However, we recommend that more plastome sequences from *Euploca*,* Tournefortia*,* Heliotropium*,* Ixorhea*, and *Myriopus* are needed to confirm the generic boundaries within Heliotropiaceae. Finally, this study will provide a baseline resource for the researchers interested in resolving the taxonomic issues within the Heliotropiaceae family.

## Methods

### Plant samples and DNA extraction

Plant Materials were collected from different regions across Saudi Arabia between March and May 2021. *E. strigosa* was collected in the Wadi Numan/Mecca region (21°19’26.7"N 40°03’22.9"E), *H. arbainense* was collected in the Wadi Al Aqiq/Medina region (24°25’26.8"N 39°33’35.7"E), and *H. longiflorum* was collected in the Al Figrah Mountains/Medina region (24°19’21.8"N 39°04’33.9"E). Samples were identified using morphological approaches and verified by Dr Dhafer Alzahrani, Department of Biological Sciences, King Abdulaziz University, Jeddah, Saudi Arabia, and then deposited in (KAUH) herbarium in King Abdulaziz University with the following voucher numbers: *E. strigosa* (MA52021), *H. arbainense* (MA62021), and *H. longiflorum* (MA72021). A DNeasy Plant Mini Kit was used to extract DNA from the plant specimens.

### Sequencing and assembly

Qualified DNA samples were sent to BGI Genomics Company in Hong Kong for library construction and sequencing using the DNBseq platform. The SOAPnuke software was used to filter the raw data [84]. Genome assembly was conducted using NOVOPlasty 4.3.1, with K-mer size equal to 33 [85]. The plastome sequence of *Heliotropium arborescens* (ON872367) was used as a reference for all three species.

### Gene annotation

All the plastomes were annotated using the GeSeq tool [86]. A map of the circular plastome was produced using OGDRAW 1.3.1 [87]. Finally, all the plastome sequences were uploaded to GenBank database with the following accession numbers: *E. strigosa* (OQ799910), *H. arbainense* (OP693483), and *H. longiflorum* (OQ756159).

### Codon usage and RNA editing sites

MEGA v.11 [88] was used to detect the codon usage in the protein-coding and tRNA sequences of the three plastomes. The PREPACT tool [89] was used to predict the RNA editing sites in the plastomes of *E. strigosa*, *H. arbainense*, and *H. longiflorum* using the BLASTX analysis mode, with a cutoff E-value of 0.8.

### Repeat analysis of plastomes

The REPuter program [90] was used to recognize the long repeats in the three plastomes. The minimal repeat sizes were set at 10 bp and the similarity among the repeat sequences was higher than 85%. The SSRs were detected using the MISA software [91] with the parameters 8, 5, 4, 3, 3, and 3 to indicate the mon, di, tri, tetra, penta, and hexa SSRs repeats.

### Characterization of the substitution rate

DNAsp v6.12.03 [92] was used to determine which genes are under selective pressure and compute the synonymous (dS) and nonsynonymous (dN) substitution rates. The protein-coding sequences of *E. strigosa*, *H. arbainense*, and *H. longiflorum* were compared to determine which genes were under selective pressure. Geneious Prime v 2023.0.4 [93] was used to extract the protein-coding sequences from the three plastome sequences.

### Genome comparison

The plastomes of *E. strigosa*, *H. arbainense*, and *H. longiflorum* were compared using the mVISTA program [94] in Shuffle-LAGAN mode. The plastomes of *H. arbainense* was used as a reference. The IRscope tool [95] was used to visualize the borders of the LSC, SSC, and IR junction positions among five Heliotropiaceae plastomes. Using DNAsp v6.12.03 [92], The sliding window analysis was performed to generate nucleotide diversity (Pi) in *E. strigosa*, *H. arbainense* and *H. longiflorum* plastomes. The step size was set to 200 bp, with an 800 bp window length.

### Phylogenetic analysis

The Phylogenetic analysis was performed based on five Heliotropiaceae plastome sequences (*E. strigosa*, *H. arbainense*, *H. arborescens*, *H. longiflorum*, and *T. montana*), 15 taxa representing four families (Boraginaceae, Cordiaceae, Ehretiaceae, and Lennoaceae) belonging to the order Boraginales, and three taxa from the Lamiaceae, Gentianaceae and Solanaceae families were used as outgroups. All the sequences were aligned using the MAFFT v.7 software [96]. The phylogenetic trees were generated using two analyses: maximum likelihood (ML) by IQ-TREE v.2.2.2.6 [97] and Bayesian inference (BI) by MrBayes v.3.2.7 [98]. First, ML analysis was conducted using 10,000 ultrafast bootstrap replicates and Modelfinder [99] was utilized to determine the substitution model (TVM + F + I + G4). Second, BI analysis was conducted using the run for 500,000 generations, sampling and printing every 250 generations, and jModelTest [100] was utilized to determine the substitution model (GTR + G).

### Electronic supplementary material

Below is the link to the electronic supplementary material.

Supplementary Material 1

## Data Availability

The datasets generated and analyzed in this study are available in the GeneBank of NCBI, and the complete plastome sequences of *Euploca strigosa*, *Heliotropium arbainense*, and *Heliotropium longiflorum* are deposited in GenBank of NCBI under the following accession numbers: *E. strigosa* (OQ799910), *H. arbainense* (OP693483), and *H. longiflorum* (OQ756159).

## Abbreviations

BI: Bayesian inference
BS: Bootstrap value
IRs: Inverted regions
LSC: Large single copy region
ML: Maximum likelihood
PP: Posterior probability
SSC: Small single copy region
SSRs: Simple sequence repeats
