# Supplementary material for: Complete plastome genomes of three medicinal heliotropiaceae species: comparative analyses and phylogenetic relationships
Source: BMC Plant Biol. 2024 Jul 10;24:654. doi: 10.1186/s12870-024-05388-8 (PMC11234707; doi:10.1186/s12870-024-05388-8)
Supplement: Supplementary file 1 — Supplementary Material 1 [file 12870_2024_5388_MOESM1_ESM.pdf]

Table S1. Base composition in the *E. strigosa*, *H. arbainense* and *H. longiflorum* plastomes.

**Species**	***E. strigosa***	***H. arbainense***	***H. longiflorum***
Genome size (bp)	155,174	154,709	154,496
IR (bp)	25,852	25,818	25,917
LSC (bp)	85,491	85,078	84,742
SSC (bp)	17,979	17,995	17,920
Total number of genes	134	134	134
rRNA	4	4	4
tRNA	30	30	30
Protein-coding genes	80	80	80
T (U) %	31.47	31.53	31.63
C %	19.27	19.23	19.04
A %	30.71	30.74	30.94
G %	18.53	18.47	18.37
Overall GC content %	37.80	37,70	37.41
GC content in LSC %	35.86	35,70	35.35
GC content in SSC %	31.84	31,73	31.13
GC content in IR %	43.10	43,10	42.95

Table S2. Gene contents in the plastomes of *E. strigosa*, *H. arbainense* and *H. longiflorum*.

Category	Gene groups	Gene Names
RNA genes	Ribosomal RNA genes (rRNA)	*rrn5* [a], *rrn4.5* [a], *rrn16* [a], *rrn23* [a]
	Transfer RNA genes (tRNA)	*trnH-GUG, trnK-UUU*[+]*, trnQ-UUG, trnS-GCU, trnG-GCC*[+]*, trnG-UCC*[+]*, trnR-UCU, trnC-GCA, trnD-GUC, trnY-GUA, trnE-UUC, trnT-GGU, trnS-UGA, trnfM- CAU, trnG-GCC, trnS-GGA, trnL-UAA*[+]*, trnT-UGU, trnF-GAA, trnV-UAC*[+]*, trnM- CAU, trnW-CCA, trnP-UGG, trnI-CAU*[+,a]*, trnL-CAA*[a]*, trnV-GAC*[a]*, trnI-GAU*[a]*,trnA-UGC*[+,a]*, trnR-ACG*[a]*, trnN-GUU*[a]*, trnL-UAG*
Ribosomal proteins	Small sub-unit of ribosome	*rps2, rps3, rps4, rps7*[a]*, rps8, rps11, rps12*[+a]*, rps14, rps15, rps16*[+]*, rps18, rps19*
Transcription Genes	Large sub-unit of ribosome	*rpl2*[+,a]*, rpl14, rpl16*[+]*, rpl20, rpl22, rpl23*[a]*, rpl32, rpl33, rpl36*
	DNA-dependent RNA polymerase	*rpoA, rpoB, rpoC1*[+]*, rpoC2*
Protein-coding genes	Photosystem I	*psaA, psaB, psaC, psaI, psaJ, ycf3*[++]
	Photosystem II	*psbA, psbB, psbC, psbD, psbE, psbF, psbH, psbI, psbJ, psbK, psbL, psbM, psbN, psbT, psbZ*
	Sub-unit of cytochrome	*petA, petB*[+]*, petD*[+]*, petG, petL, petN*
	Sub-unit of synthase	*atpA, atpB, atpE, atpF*[+]*, atpH, atpI*
	Large sub-unit of rubisco	*rbcL*
	NADH dehydrogenase	*ndhA*[+]*, ndhB*[+a]*, ndhC, ndhD, ndhE, ndhF, ndhG, ndhH, ndhI, ndhJ, ndhK*
	ATP-dependent protease subunit P	*clpP*[++]
Other genes	Cp envelope membrane protein	*cemA*
	Maturase	*matK*
	Sub-unit acetyl-coA carboxylase	*accD*
	C-type cytochrome systhesis	*ccsA*
	Translation initiation factor	*infA*
	Hypothetical proteins	*ycf2*[a]*, ycf4, ycf15*[a]
	Component of TIC complex	*ycf1*[a]

*** +** Gene with one intron, **++** Gene with two introns and **a** Gene with multiple copies. *trnG-GCC* gene was absent in *E. strigosa* and *H. longiflorum.* while *trnG-UCC* gene was absent in *H. arbainense.*

Table S3. Length of introns and exons in the of *E. strigosa*, *H. arbainense* and *H. longiflorum* plastomes.

Gene	Location	Exon I (bp)			Intron I (bp)			Exon II (bp)			Intron II (bp)			Exon III (bp)		
		E. s	***H. a***	***H. l***	***E. s***	***H. a***	***H. l***	***E. s***	***H. a***	***H. l***	***E. s***	***H. a***	***H. l***	***E. s***	***H. a***	***H. l***
trnK-UUU	LSC	34	34	34	2487	2488	2472	36	36	36						
rps16	LSC	226	226	226	874	861	892	39	39	39						
trnG-GCC	LSC	*	22	*	*	667	*	*	47	*						
trnG-UCC	LSC	22	*	22	668	*	699	47	*	47						
atpF	LSC	410	410	410	704	703	695	143	143	143						
rpoC1	LSC	1612	1612	1612	757	754	764	429	450	429						
ycf3	LSC	154	154	154	747	739	767	227	227	227	728	726	751	123	123	123
trnL-UAA	LSC	34	34	34	500	502	509	49	49	49						
trnV-UAC	LSC	-	36	-	-	599	-	-	37	-						
clpP1	LSC	227	227	233	621	611	597	291	291	291	771	765	801	70	70	70
petB	LSC	5	5	5	782	790	696	641	641	641						
petD	LSC	7	7	7	681	693	687	474	474	474						
rpl16	LSC	398	398	398	1059	1059	787	8	8	8						
rpl2	IR	433	433	433	660	660	658	390	390	390						
ndhB	IR	757	755	757	678	678	677	774	776	774						
rps12	IR	25	25	25	534	534	535	231	231	231						
trnI-GAU	IR	36	36	36	945	944	948	34	34	34						
trnA-UGC	IR	37	37	37	811	811	817	34	34	34						
ndhA	SSC	538	538	538	1102	1105	1083	552	552	552						

Table S4. Codon-anticodon recognition patterns and codon usage of the *E. strigosa* plastome.

Codon	Amino Acid	RSCU	tRNA	Codon	Amino Acid	RSCU	tRNA
UUU	Phe	1.18	trnF-GAA	UAU(Y)	Tyr	1.32	trnY-GUA
UUC	Phe	0.82		UAC(Y)	Tyr	0.68	
UUA	Leu	1.3	trnL-UAA	UAA(*)	Stop	0.99	
UUG	Leu	1.31	trnL-CAA	UAG(*)	Stop	0.94	
CUU	Leu	1.14	trnL-UAG	CAU(H)	His	1.29	trnH-GUG
CUC	Leu	0.61		CAC(H)	His	0.71	
CUA	Leu	0.97		CAA(Q)	Gln	1.24	trnQ-UUG
CUG	Leu	0.66		CAG(Q)	Gln	0.76	
AUU	Ile	1.24	trnI-GAU	AAU(N)	Asn	1.31	trnN-GUU
AUC	Ile	0.82		AAC(N)	Asn	0.69	
AUA	Ile	0.93	trnI-CAU	AAA(K)	Lys	1.22	trnK-UUU
AUG	Met	1	trnM-CAU	AAG(K)	Lys	0.78	
GUU	Val	1.36	trnV-GAC	GAU(D)	Asp	1.36	trnD-GUC
GUC	Val	0.65		GAC(D)	Asp	0.64	
GUA	Val	1.24		GAA(E)	Glu	1.3	trnE-UUC
GUG	Val	0.75	trnV-UAC	GAG(E)	Glu	0.7	
UCU	Ser	1.24	trnS-GGA	UGU(C)	Cys	1.06	trnC-GCA
UCC	Ser	0.93		UGC(C)	Cys	0.94	
UCA	Ser	1.43		UGA(*)	Stop	1.07	
UCG	Ser	0.84	trnS-UGA	UGG(W)	Trp	1	trnW-CCA
CCU	Pro	1.01	trnP-UGG	CGU(R)	Arg	0.55	trnR-ACG
CCC	Pro	0.81		CGC(R)	Arg	0.4	trnR-UCU
CCA	Pro	1.25		CGA(R)	Arg	0.91	
CCG	Pro	0.93		CGG(R)	Arg	0.84	
ACU	Thr	1.08		AGA(R)	Arg	1.98	
ACC	Thr	0.82		AGG(R)	Arg	1.32	
ACA	Thr	1.33	trnT-GGU	AGU(S)	Ser	0.84	trnS-GCU
ACG	Thr	0.77	trnT-UGU	AGC(S)	Ser	0.73	
GCU	Ala	1.31	trnA-UGC	GGU(G)	Gly	0.96	trnG-GCC
GCC	Ala	0.83		GGC(G)	Gly	0.63	
GCA	Ala	1.14		GGA(G)	Gly	1.32	
GCG	Ala	0.72		GGG(G)	Gly	1.09	trnG-UCC

Table S5. Codon-anticodon recognition patterns and codon usage of the *H. arbainense* plastome.

Codon	Amino Acid	RSCU	tRNA	Codon	Amino Acid	RSCU	tRNA
UUU	Phe	1.15	trnF-GAA	UAU(Y)	Tyr	1.35	trnY-GUA
UUC	Phe	0.85		UAC(Y)	Tyr	0.65	
UUA	Leu	1.33	trnL-UAA	UAA(*)	Stop	0.94	
UUG	Leu	1.32	trnL-CAA	UAG(*)	Stop	1.06	
CUU	Leu	1.15	trnL-UAG	CAU(H)	His	1.33	trnH-GUG
CUC	Leu	0.65		CAC(H)	His	0.67	
CUA	Leu	0.91		CAA(Q)	Gln	1.29	trnQ-UUG
CUG	Leu	0.63		CAG(Q)	Gln	0.71	
AUU	Ile	1.2	trnI-GAU	AAU(N)	Asn	1.35	trnN-GUU
AUC	Ile	0.8		AAC(N)	Asn	0.65	
AUA	Ile	1	trnI-CAU	AAA(K)	Lys	1.25	trnK-UUU
AUG	Met	1	trnM-CAU	AAG(K)	Lys	0.75	
GUU	Val	1.41	trnV-GAC	GAU(D)	Asp	1.43	trnD-GUC
GUC	Val	0.63		GAC(D)	Asp	0.57	
GUA	Val	1.18		GAA(E)	Glu	1.37	trnE-UUC
GUG	Val	0.78	trnV-UAC	GAG(E)	Glu	0.63	
UCU	Ser	1.48	trnS-GGA	UGU(C)	Cys	1.22	trnC-GCA
UCC	Ser	0.89		UGC(C)	Cys	0.78	
UCA	Ser	1.33		UGA(*)	Stop	1	
UCG	Ser	0.82	trnS-UGA	UGG(W)	Trp	1	trnW-CCA
CCU	Pro	1.09	trnP-UGG	CGU(R)	Arg	0.68	trnR-ACG
CCC	Pro	0.81		CGC(R)	Arg	0.37	trnR-UCU
CCA	Pro	1.26		CGA(R)	Arg	1.06	
CCG	Pro	0.83		CGG(R)	Arg	0.79	
ACU	Thr	1.22		AGA(R)	Arg	1.97	
ACC	Thr	0.77		AGG(R)	Arg	1.13	
ACA	Thr	1.32	trnT-GGU	AGU(S)	Ser	0.94	trnS-GCU
ACG	Thr	0.69	trnT-UGU	AGC(S)	Ser	0.54	
GCU	Ala	1.39	trnA-UGC	GGU(G)	Gly	1.04	trnG-GCC
GCC	Ala	0.75		GGC(G)	Gly	0.55	
GCA	Ala	1.16		GGA(G)	Gly	1.35	
GCG	Ala	0.7		GGG(G)	Gly	1.05	trnG-UCC

Table S6. Codon-anticodon recognition patterns and codon usage of the *H. longiflorum* plastome.

Codon	Amino Acid	RSCU	tRNA	Codon	Amino Acid	RSCU	tRNA
UUU	Phe	1.2	trnF-GAA	UAU(Y)	Tyr	1.34	trnY-GUA
UUC	Phe	0.8		UAC(Y)	Tyr	0.66	
UUA	Leu	1.37	trnL-UAA	UAA(*)	Stop	1.05	
UUG	Leu	1.35	trnL-CAA	UAG(*)	Stop	0.94	
CUU	Leu	1.14	trnL-UAG	CAU(H)	His	1.43	trnH-GUG
CUC	Leu	0.66		CAC(H)	His	0.57	
CUA	Leu	0.85		CAA(Q)	Gln	1.3	trnQ-UUG
CUG	Leu	0.63		CAG(Q)	Gln	0.7	
AUU	Ile	1.24	trnI-GAU	AAU(N)	Asn	1.35	trnN-GUU
AUC	Ile	0.78		AAC(N)	Asn	0.65	
AUA	Ile	0.97	trnI-CAU	AAA(K)	Lys	1.25	trnK-UUU
AUG	Met	1	trnM-CAU	AAG(K)	Lys	0.75	
GUU	Val	1.33	trnV-GAC	GAU(D)	Asp	1.4	trnD-GUC
GUC	Val	0.74		GAC(D)	Asp	0.6	
GUA	Val	1.2		GAA(E)	Glu	1.29	trnE-UUC
GUG	Val	0.72	trnV-UAC	GAG(E)	Glu	0.71	
UCU	Ser	1.44	trnS-GGA	UGU(C)	Cys	1.15	trnC-GCA
UCC	Ser	0.9		UGC(C)	Cys	0.85	
UCA	Ser	1.24		UGA(*)	Stop	1.01	
UCG	Ser	0.86	trnS-UGA	UGG(W)	Trp	1	trnW-CCA
CCU	Pro	1.1	trnP-UGG	CGU(R)	Arg	0.71	trnR-ACG
CCC	Pro	0.85		CGC(R)	Arg	0.33	trnR-UCU
CCA	Pro	1.15		CGA(R)	Arg	0.97	
CCG	Pro	0.89		CGG(R)	Arg	0.72	
ACU	Thr	1.16		AGA(R)	Arg	2	
ACC	Thr	0.82		AGG(R)	Arg	1.27	
ACA	Thr	1.26	trnT-GGU	AGU(S)	Ser	0.93	trnS-GCU
ACG	Thr	0.76	trnT-UGU	AGC(S)	Ser	0.65	
GCU	Ala	1.35	trnA-UGC	GGU(G)	Gly	0.95	trnG-GCC
GCC	Ala	0.82		GGC(G)	Gly	0.6	
GCA	Ala	1.08		GGA(G)	Gly	1.37	
GCG	Ala	0.76		GGG(G)	Gly	1.08	trnG-UCC

Table S7. Predicted RNA editing site in the *E. strigosa* plastome.

Gene	Nucleotide position	Amino acid position	Triplet position within codon	Base conversion	Codon change	Amino acid conversion
matK	655	219	1	C→U	CAU→UAU	H→Y
atpF	92	31	2	C→U	CCA→CUA	P→L
rps2	248	83	2	C→U	UCA→UUA	S→L
rpoC1	65	22	2	C→U	UCA→UUA	S→L
rpoB	338	113	2	C→U	UCU→UUU	S→F
	473	158	2	C→U	UCA→UUA	S→L
	551	184	2	C→U	UCA→UUA	S→L
	2432	811	2	C→U	UCA→UUA	S→L
psbZ	50	17	2	C→U	UCA→UUA	S→L
rps14	80	27	2	C→U	UCA→UUA	S→L
rps14	149	50	2	C→U	CCA→CUA	P→L
psbL	2	1	2	C→U	ACG→AUG	T→M
psbE	214	72	1	C→U	CCU→UCU	P→S
petB	611	204	2	C→U	CCA→CUA	P→L
rpoA	842	281	2	C→U	UCA→UUA	S→L
rpl23	71	24	2	C→U	UCU→UUU	S→F
	89	30	2	C→U	UCA→UUA	S→L
ndhB	53	18	2	C→U	UCA→UUA	S→L
	59	20	2	C→U	UCA→UUA	S→L
	95	32	2	C→U	UCA→UUA	S→L
	149	50	2	C→U	UCA→UUA	S→L
	467	156	2	C→U	CCA→CUA	P→L
	586	196	1	C→U	CAU→UAU	H→Y
	704	235	2	C→U	CCA→CUA	P→L
	737	246	2	C→U	CCA→CUA	P→L
ndhF	290	97	2	C→U	UCA→UUA	S→L
ndhD	2	1	2	C→U	ACG→AUG	T→M
	599	200	2	C→U	UCA→UUA	S→L
	674	225	2	C→U	UCG→UUG	S→L
	878	293	2	C→U	UCA→UUA	S→L
	1298	433	2	C→U	UCA→UUA	S→L
ndhA	341	114	2	C→U	UCA→UUA	S→L
	409	137	1	C→U	CCU→UCU	P→S
	521	174	2	C→U	UCC→UUC	S→F

Table S8. Predicted RNA editing site in the *H. arbainense* and *H. longiflorum* plastomes.

Gene	Nucleotide position	Amino acid position	Triplet position within codon	Base conversion	Codon change	Amino acid conversion
matK	655	219	1	C→U	CAU→UAU	H→Y
atpF	92	31	2	C→U	CCA→CUA	P→L
rps2	248	83	2	C→U	UCA→UUA	S→L
rpoC1	65	22	2	C→U	UCA→UUA	S→L
rpoB	338	113	2	C→U	UCU→UUU	S→F
	473	158	2	C→U	UCA→UUA	S→L
	551	184	2	C→U	UCA→UUA	S→L
	2432	811	2	C→U	UCA→UUA	S→L
psbZ	50	17	2	C→U	UCA→UUA	S→L
rps14	80	27	2	C→U	UCA→UUA	S→L
	149	50	2	C→U	CCA→CUA	P→L
psbE	214	72	1	C→U	CCU→UCU	P→S
petB	611	204	2	C→U	CCA→CUA	P→L
rpoA	200	67	2	C→U	UCU→UUU	S→F
rpoA	842	281	2	C→U	UCA→UUA	S→L
rpl23	71	24	2	C→U	UCU→UUU	S→F
	89	30	2	C→U	UCA→UUA	S→L
ndhB	53	18	2	C→U	UCA→UUA	S→L
	59	20	2	C→U	UCA→UUA	S→L
	95	32	2	C→U	UCA→UUA	S→L
	149	50	2	C→U	UCA→UUA	S→L
	467	156	2	C→U	CCA→CUA	P→L
	586	196	1	C→U	CAU→UAU	H→Y
	704	235	2	C→U	CCA→CUA	P→L
	737	246	2	C→U	CCA→CUA	P→L
ndhF	290	97	2	C→U	UCA→UUA	S→L
ndhD	2	1	2	C→U	ACG→AUG	T→M
	599	200	2	C→U	UCA→UUA	S→L
	878	293	2	C→U	UCA→UUA	S→L
	1298	433	2	C→U	UCA→UUA	S→L
ndhA	341	114	2	C→U	UCA→UUA	S→L
	409	137	1	C→U	CCU→UCU	P→S
	521	174	2	C→U	UCC→UUC	S→F

*The Prediction of RNA editing site in *psbE* gene was only found in *H. longiflorum*.

Table S9.Repeat sequences present in the *E. strigosa* plastome.

SN	Repeat Size	Repeat Position 1	Repeat Type	Repeat Location 1	Repeat Position 2	Repeat Location 2	E-Value
**1**	44	75616	P	IGS	75616	IGS	0 2.19e-17
**2**	40	9824	P	IGS	9824	IGS	0 5.60e-15
**3**	29	8177	P	IGS	45753	trnS-GGA	0 2.35e-08
**4**	29	114600	F	ndhF	114626	IGS	0 2.35e-08
**5**	26	89128	P	ycf2	89128	ycf2	0 1.50e-06
**6**	26	89128	F	ycf2	151511	ycf2	0 1.50e-06
**7**	26	121786	P	ndhA - intron	121786	ndhA - intron	0 1.50e-06
**8**	26	151511	P	ycf2	151511	ycf2	0 1.50e-06
**9**	24	32169	F	IGS	32191	IGS	0 2.41e-05
**19**	24	39503	F	psaB	41727	psaA	0 2.41e-05
**11**	24	55945	F	IGS	55968	IGS	0 2.41e-05
**12**	24	126151	P	ycf1	126151	ycf1	0 2.41e-05
**13**	23	27891	F	IGS	27914	IGS	0 9.62e-05
**14**	22	9750	F	trnG-UCC	37284	trnG-GCC	0 3.85e-04
**15**	22	95208	P	IGS	95234	IGS	0 3.85e-04
**16**	22	95208	F	IGS	145409	IGS	0 3.85e-04
**17**	22	95234	F	IGS	145435	IGS	0 3.85e-04
**18**	22	145409	P	IGS	145435	IGS	0 3.85e-04
**19**	21	241	P	IGS	291	IGS	0 1.54e-03
**20**	21	6805	R	IGS	6805	IGS	0 1.54e-03
**21**	21	8182	F	trnS-GCU	36364	trnS-UGA	0 1.54e-03
**22**	21	30305	P	IGS	30336	IGS	0 1.54e-03
**23**	21	36364	P	trnS-UGA	45756	trnS-GGA	0 1.54e-03
**24**	21	37498	F	trnfM-CAU	67949	trnP-UGG	0 1.54e-03
**25**	21	92777	F	ycf2	92795	ycf2	0 1.54e-03
**26**	21	92777	P	ycf2	147849	ycf2	0 1.54e-03
**27**	21	92795	P	ycf2	147867	ycf2	0 1.54e-03
**28**	21	99797	F	IGS	121412	ndhA - intron	0 1.54e-03
**29**	21	121412	P	ndhA - intron	140847	IGS	0 1.54e-03
**30**	21	147849	F	ycf2	147867	ycf2	0 1.54e-03
**31**	20	8407	F	IGS	8425	IGS	0 6.16e-03
**32**	20	29669	F	IGS	29688	IGS	0 6.16e-03
**33**	20	31977	P	IGS	31977	IGS	0 6.16e-03
**34**	20	50956	R	ndhC	50956	ndhC	0 6.16e-03
**35**	20	52906	P	trnV-UAC	104513	trnA-UGC	0 6.16e-03
**36**	20	52906	F	trnV-UAC	136132	trnA-UGC	0 6.16e-03
**37**	19	2818	R	trnK-UUU - intron	114895	IGS	0 2.46e-02
**38**	19	6304	R	IGS	46714	IGS	0 2.46e-02
**39**	19	6306	F	IGS	6326	IGS	0 2.46e-02
**40**	19	15199	R	IGS	15199	IGS	0 2.46e-02
**41**	19	16768	P	rpoC2	60777	IGS	0 2.46e-02
**42**	19	43091	F	IGS	111358	ycf1	0 2.46e-02
**43**	19	51380	F	IGS	51397	IGS	0 2.46e-02
**44**	19	55702	C	IGS	92182	ycf2	0 2.46e-02
**45**	19	55702	R	IGS	148464	ycf2	0 2.46e-02
**46**	19	61751	F	IGS	72876	clpP1 - intron	0 2.46e-02
**47**	19	118218	P	IGS	118243	IGS	0 2.46e-02
**48**	18	8620	F	IGS	28888	IGS	0 9.85e-02
**49**	18	31641	P	IGS	45149	ycf3 - intron	0 9.85e-02

Table S10. Repeat sequences present in the *H. arbainense* plastome.

SN	Repeat Size	Repeat Position 1	Repeat Type	Repeat Location 1	Repeat Position 2	Repeat Location 2	E-Value
**1**	40	9555	P	IGS	9555	IGS	0 5.57e-15
**2**	29	7942	P	IGS	45552	trnS-GGA	0 2.34e-08
**3**	26	88679	P	ycf2	88679	ycf2	0 1.49e-06
**4**	26	88679	F	ycf2	151082	ycf2	0 1.49e-06
**5**	26	121326	P	ndhA - Intron	121326	ndhA - Intron	0 1.49e-06
**6**	26	151082	P	ycf2	151082	ycf2	0 1.49e-06
**7**	25	26984	F	IGS	27008	IGS	0 5.98e-06
**8**	24	39337	F	psaB	41561	psaA	0 2.39e-05
**9**	24	111676	R	ndhF	111676	ndhF	0 2.39e-05
**19**	22	9486	F	trnG-GCC	37121	trnG-GCC	0 3.83e-04
**11**	22	82589	P	rpl16 - Intron	82589	rpl16 - Intron	0 3.83e-04
**12**	22	94770	P	IGS	94796	IGS	0 3.83e-04
**13**	22	94770	F	IGS	144969	IGS	0 3.83e-04
**14**	22	94796	F	IGS	144995	IGS	0 3.83e-04
**15**	22	144969	P	IGS	144995	IGS	0 3.83e-04
**16**	21	101	R	IGS	101	IGS	0 1.53e-03
**17**	21	286	P	IGS	336	IGS	0 1.53e-03
**18**	21	7947	F	trnS-GCU	36169	trnS-UGA	0 1.53e-03
**19**	21	29990	P	IGS	30021	IGS	0 1.53e-03
**20**	21	33245	F	IGS	33266	IGS	0 1.53e-03
**21**	21	36169	P	trnS-UGA	45555	trnS-GGA	0 1.53e-03
**22**	21	37331	F	trnfM-CAU	67517	trnP-UGG	0 1.53e-03
**23**	21	92334	F	ycf2	92352	ycf2	0 1.53e-03
**24**	21	92334	P	ycf2	147414	ycf2	0 1.53e-03
**25**	21	92352	P	ycf2	147432	ycf2	0 1.53e-03
**26**	21	99359	F	IGS	120943	ndhA - Intron	0 1.53e-03
**27**	21	120943	P	ndhA - Intron	140407	IGS	0 1.53e-03
**28**	21	147414	F	ycf2	147432	ycf2	0 1.53e-03
**29**	20	31657	P	IGS	31657	IGS	0 6.12e-03
**30**	20	50676	R	ndhC	50676	ndhC	0 6.12e-03
**31**	20	52593	P	trnV-UAC	104067	trnA-UGC	0 6.12e-03
**32**	20	52593	F	trnV-UAC	135700	trnA-UGC	0 6.12e-03
**33**	19	119	R	IGS	115293	ccsA	0 2.45e-02
**34**	19	14927	R	atpI	14927	atpI	0 2.45e-02
**35**	19	30148	F	IGS	30162	IGS	0 2.45e-02
**36**	19	32524	C	IGS	55519	IGS	0 2.45e-02
**37**	19	42925	F	IGS	110911	ndhF	0 2.45e-02
**38**	19	75154	P	IGS	75179	IGS	0 2.45e-02
**39**	19	111683	R	ndhF	111683	ndhF	0 2.45e-02
**40**	19	116195	R	IGS	116195	IGS	0 2.45e-02
**41**	19	117758	P	IGS	117783	IGS	0 2.45e-02
**42**	19	121239	F	ndhA - Intron	121258	ndhA - Intron	0 2.45e-02
**43**	18	6483	F	IGS	47194	IGS	0 9.80e-02
**44**	18	16475	P	rpoC2	60408	IGS	0 9.80e-02
**45**	18	27899	R	IGS	27899	IGS	0 9.80e-02
**46**	18	36364	F	IGS	36382	IGS	0 9.80e-02
**47**	18	47349	R	IGS	47349	IGS	0 9.80e-02
**48**	18	57348	F	IGS	57368	IGS	0 9.80e-02
**49**	18	63960	P	IGS	108551	IGS	0 9.80e-02

Table S11. Repeat sequences present in the *H. longiflorum* plastome.

SN	Repeat Size	Repeat Position 1	Repeat Type	Repeat Location 1	Repeat Position 2	Repeat Location 2	E-Value
**1**	48	75187	P	IGS	75187	IGS	8.47E-20
**2**	40	99085	F	IGS	120662	ndhA - Intron	5.55E-15
**3**	40	120662	P	ndhA - Intron	140113	IGS	5.55E-15
**4**	38	113438	F	IGS	113458	IGS	8.88E-14
**5**	29	8381	P	IGS	45931	trnS-GGA	2.33E-08
**6**	26	39349	F	psaB	41573	psaA	1.49E-06
**7**	26	81405	R	IGS	81405	IGS	1.49E-06
**8**	26	88412	P	ycf2	88412	ycf2	1.49E-06
**9**	26	88412	F	ycf2	150800	ycf2	1.49E-06
**19**	26	150800	P	ycf2	150800	ycf2	1.49E-06
**11**	23	4297	R	IGS	4297	IGS	9.54E-05
**12**	23	33269	F	IGS	33290	IGS	9.54E-05
**13**	23	64638	F	IGS	64660	IGS	9.54E-05
**14**	22	9660	F	trnG-UCC	37145	trnG-GCC	3.82E-04
**15**	22	55554	C	IGS	81411	IGS	3.82E-04
**16**	22	94504	P	IGS	94530	IGS	3.82E-04
**17**	22	94504	F	IGS	144686	IGS	3.82E-04
**18**	22	94530	F	IGS	144712	IGS	3.82E-04
**19**	22	144686	P	IGS	144712	IGS	3.82E-04
**20**	21	98	F	IGS	119	IGS	1.53E-03
**21**	21	8386	F	trnS-GCU	36185	trnS-UGA	1.53E-03
**22**	21	33047	R	IGS	33047	IGS	1.53E-03
**23**	21	36185	P	trnS-UGA	45934	trnS-GGA	1.53E-03
**24**	21	37346	F	trnfM-CAU	67578	trnP-UGG	1.53E-03
**25**	21	55554	P	IGS	121069	ndhA - Intron	1.53E-03
**26**	21	81411	R	IGS	121069	ndhA - Intron	1.53E-03
**27**	21	92061	F	ycf2	92079	ycf2	1.53E-03
**28**	21	92061	P	ycf2	147138	ycf2	1.53E-03
**29**	21	92079	P	ycf2	147156	ycf2	1.53E-03
**30**	21	147138	F	ycf2	147156	ycf2	1.53E-03
**31**	20	28959	F	IGS	28979	IGS	6.11E-03
**32**	20	31773	P	IGS	31773	IGS	6.11E-03
**33**	20	51180	R	ndhC	51180	ndhC	6.11E-03
**34**	20	55554	P	IGS	81405	IGS	6.11E-03
**35**	20	81405	F	IGS	121070	ndhA - Intron	6.11E-03
**36**	20	125563	P	ycf1	125563	ycf1	6.11E-03
**37**	19	4336	P	IGS	121074	ndhA - Intron	2.44E-02
**38**	19	12278	P	atpF - Intron	81953	IGS	2.44E-02
**39**	19	14971	R	atpI	14971	atpI	2.44E-02
**40**	19	16241	P	IGS	45334	IGS	2.44E-02
**41**	19	18418	R	rpoC2	32902	IGS	2.44E-02
**42**	19	50997	R	IGS	127061	ycf1	2.44E-02
**43**	18	224	P	IGS	270	IGS	9.77E-02
**44**	18	4603	F	IGS	72191	clpP1 - Intron	9.77E-02
**45**	18	8451	F	trnS-GCU	36251	trnS-UGA	9.77E-02
**46**	18	15300	P	IGS	126815	ycf1	9.77E-02
**47**	18	22832	C	rpoC1 - Intron	51796	IGS	9.77E-02
**48**	18	24721	C	rpoB	28778	IGS	9.77E-02
**49**	18	31227	C	IGS	114405	IGS	9.77E-02

Table S12. Simple sequence repeats in the plastome of *E. strigosa*.

Repeat	Length (bp)	Number	Start position
A	8	27	1568; 8628; 9183; 12201; 15530;18806; 21256; 22463; 28896; 29962; 38134; 43104; 46875; 47521; 55855; 63636; 65639; 72077; 72539; 76746; 83953; 89780; 98163; 115759; 121630; 129336; 155087
	9	20	4214; 4524; 8628; 30975; 37113; 49012; 55855; 69256; 69770; 79202; 90954; 109523; 114779; 118269; 136763; 140710; 141794; 145694; 155087; 155162
	10	6	8150; 49151; 60788; 64721; 94771; 115214
	11	6	86; 36591; 44559; 67287; 82441; 114169
	12	2	48147; 66831
	13	1	83246
	16	1	13640
C	8	1	16769
	9	1	37402
G	8	1	63098
	10	1	78232
T	8	34	2166; 4524; 7512; 8628; 15530; 16472; 22771; 26247; 26358; 28134; 29631; 43104; 49361; 50771; 51273; 51579; 52350; 61029; 62110; 64373; 71848; 76618; 83246; 84099; 85504; 111323; 114998; 116091; 117281; 127092; 129096; 142496; 150879
	9	27	7908; 16769; 44145; 49012; 55254; 59752; 67414; 69770; 75377; 78232; 84809; 85259; 85504; 85579; 94964; 98864; 99948; 103895; 114169; 116577; 121630; 124352; 127321; 127400; 127695; 131135; 149704
	10	6	30604; 55254; 57971; 70445; 71848; 145886
	11	7	9933; 13084; 32719; 52053; 60452; 61756; 72881
	12	1	66370
	13	3	12425; 18663; 81912
	17	1	32874
AT	5	4	20027; 31110; 86198; 154459
	6	1	6103
TA	5	2	32184; 32215
ATA	4	2	45525; 67849
TAT	4	1	64721
AAAC	3	1	69256
TCTT	3	1	30335

Table S13. Simple sequence repeats in the plastome of *H. arbainense*.

Repeat	Length (bp)	Number	Start position
A	8	26	108; 1622; 8364; 15258; 18512; 20977; 22184; 23478; 28602; 29635; 46645; 60419; 63262; 64352; 65238; 68831; 69340; 72082; 72102; 89331; 97725; 115300; 117809; 128905; 136331; 145260
	9	20	108; 4569; 30682; 36899; 36958; 37967; 44372; 47285; 47913; 48907; 65968; 82805; 83513; 90511; 109076; 114742; 121220; 140270; 141354; 154647
	10	11	4263; 4569; 7921; 8364; 31826; 55520; 66880; 76282; 78710; 94328; 113732
	11	2	4569; 55520
	13	3	13380; 114315; 154647
	15	1	66421
C	8	2	16475; 37239
G	10	1	77777
T	8	42	4569; 7292; 7495; 8364; 16190; 25965; 26076; 27847; 27912; 31343; 43007; 48771; 49110; 50491; 50993; 51273; 60658; 61186; 61733; 71395; 76156; 77777; 78710; 82805; 83513; 84818; 94521; 103450; 110876; 113732; 114315; 115632; 116821; 119478; 124729; 125712; 126519; 126640; 128659; 142056; 150450
	9	25	7689; 15258; 16475; 29271; 30303; 43958; 51747; 59406; 67006; 70014; 71395;84368; 85094; 98426; 99510; 114541; 123886; 124915; 126869; 126948; 127243; 130704; 149269
	10	10	9664; 12151; 30817; 47913; 52042; 54941; 60101; 72425; 74913; 145451
	11	3	12813; 57634; 65968
	12	3	22489; 32523; 61387
	13	3	18369; 85094; 116199
	14	2	32679; 81448
AT	5	4	19742; 30817; 85749; 154030
TA	6	1	32009
AAAC	3	1	68831
ATAA	3	1	83358
TCTT	3	1	30020
TTAA	4	1	82593
AATAA	3	1	130413
TTTAT	3	1	109360

Table S14. Simple sequence repeats in the plastome of *H. longiflorum*.

Repeat	Length (bp)	Number	Start position
A	8	23	4510; 8362; 18564; 22234; 28551; 31238; 36898; 37979; 42902; 43507; 44475; 48182; 63517; 65531; 66504; 66822; 80329; 83220; 108775; 111364; 114091; 136026; 154430
	9	19	4158; 4510; 4679; 6252; 29603; 36898; 47042; 64576; 64973; 72075; 72201; 74990; 76356; 90238; 94054; 97463; 130410; 141066; 154430
	10	9	3699; 7056; 8702; 45342; 47888; 60171; 69325; 81959; 117539
	11	5	9253; 15303; 36425; 78723; 82549
	12	3	13501; 32215; 81959
	13	1	22832
	14	1	4283
	16	1	55553
C	9	1	99171
	12	1	100765
	13	1	36898
G	9	2	43507; 140060
	12	1	138463
T	8	22	7281; 26025; 26136; 27215; 27870; 29453; 32215; 36047; 42902; 45342; 50995; 64576; 66242; 67086; 84769; 103206; 115355; 116552; 121068; 126344; 128363; 130410;
	9	25	4510; 10016; 16530; 22832; 30248; 48300; 49225; 61648; 61674; 66242; 71435; 74854; 83724; 84523; 84769; 98165; 108775; 113228; 123606; 125380; 126573; 126821; 141768; 145177; 148993
	10	10	2126; 4283; 8129; 12283; 16244; 47607; 54997; 59625; 77733; 114416
	11	7	8702; 44064; 57815; 72314; 78968; 126821; 126958
	12	3	51797; 72314; 115930
	13	2	18421; 32907
	14	2	81412; 121068
AT	5	3	14480; 19794; 48300
TA	5	1	8543
GCT	4	1	57152
TTC	4	1	35839
AAAC	3	1	68897
AAAT	3	1	49558
TAAA	3	1	4283
TTTA	3	1	51600
TTTG	3	1	5460
TTCTTT	3	1	44064
